# CRISPR-Cas9 effectors facilitate generation of single-sex litters and sex-specific phenotypes

**DOI:** 10.1038/s41467-021-27227-2

**Published:** 2021-12-03

**Authors:** Charlotte Douglas, Valdone Maciulyte, Jasmin Zohren, Daniel M. Snell, Shantha K. Mahadevaiah, Obah A. Ojarikre, Peter J. I. Ellis, James M. A. Turner

**Affiliations:** 1 Sex Chromosome Biology Laboratory, The Francis Crick Institute, London, UK; 2 School of Biosciences, University of Kent, Kent, UK

**Keywords:** Genetics, Developmental biology, Genetic engineering, CRISPR-Cas systems

## Abstract

Animals are essential genetic tools in scientific research and global resources in agriculture. In both arenas, a single sex is often required in surplus. The ethical and financial burden of producing and culling animals of the undesired sex is considerable. Using the mouse as a model, we develop a synthetic lethal, bicomponent CRISPR-Cas9 strategy that produces male- or female-only litters with one hundred percent efficiency. Strikingly, we observe a degree of litter size compensation relative to control matings, indicating that our system has the potential to increase the yield of the desired sex in comparison to standard breeding designs. The bicomponent system can also be repurposed to generate postnatal sex-specific phenotypes. Our approach, harnessing the technological applications of CRISPR-Cas9, may be applicable to other vertebrate species, and provides strides towards ethical improvements for laboratory research and agriculture.

## Abstract (web summary)

In areas such as animal research and agriculture a single sex is often required in abundance, leading to wasted resources and ethical considerations. Here the authors develop a CRISPR/Cas9 mediated synthetic lethal system that enables the production of single sex offspring that can be repurposed for use in multiple organisms.

## Introduction

Animals and animal products are used globally, and ethical discussions regarding animal usage are ongoing. Applications that specifically require females (XX) or males (XY) are especially contentious, because the unrequired sex is generated needlessly and must be culled. In laboratory research, the “Replacement, Reduction and Refinement” (3Rs) global guidelines^1^ promote efficient animal use, but shortcomings remain. In Great Britain, over 1.8 million laboratory animals were used for breeding and tissue supply alone^2^. Although good experimental design should, where possible, consider both sexes^3,4^, many topics necessitate a specific sex. These include oogenesis, spermatogenesis, placental biology, breast, prostate and other sex-limited cancers, sex steroid hormone pathways, Y-chromosome biology and X-chromosome inactivation (XCI)^5^. In agriculture, the dairy industry requires females, and as a result, each year around 95,000 male calves are culled in the UK^6^, 200,000 in Germany^7^ and 500,000 in Australia^8^. In contrast, in the beef industry males are favoured for their faster, leaner growth and more efficient feed conversion ratios. In the pig breeding industry, accumulation of androstenone and skatole in post-pubertal males creates an offensive meat flavour (boar taint). Consequently, males can only be used for meat production if they are castrated or slaughtered prior to puberty, with significant ethical and economic implications^9^. Methods to generate single-sex litters in research and agriculture are therefore urgently needed.

Flow sorting of X- and Y-bearing sperm is employed to an extent in cattle, albeit with an associated reduction in fertility^10,11^. However, sperm sexing is not feasible in other livestock species such as pigs, or in laboratory mice. Embryo selection via lethality of male or female embryos offers an alternative and more widely applicable method to control offspring sex ratio. This selection can be achieved by a synthetic lethal strategy, in which a dormant suicide gene carried on the paternal X or Y chromosome is inherited in a sex-specific manner. This suicide gene is activated by a second trigger gene contributed by the mother. Embryos inheriting both genes die, while those carrying only one of the genes survive.

Variations on this type of bicomponent CRISPR-Cas9 approach have been used in silkworms^12^, *Drosophila*^13^, mosquitos^14^ and zebrafish^15^, but have never been efficiently deployed for sex ratio control in mammals. A major hurdle in mammals is posed by the mammalian X and Y chromosome: transgenes on the X chromosome are susceptible to silencing by XCI^16^, and those on the Y chromosome are repressed by its heterochromatic state^17,18^. The generation of male-only mouse litters has not been described. Generation of female-only mouse litters was attempted, by combining an autosome-encoded Cas9 with a Y-linked single-guide RNA (sgRNA) transgene targeting three essential developmental genes^19^. However, this approach produced a female skew rather than female-only litters, with some surviving male pups exhibiting severe developmental defects resulting from Cas9-induced mutations.

A challenge also arises when studying the sex-specific effects of mutations that have a universally harmful postnatal phenotype, for example when assessing the reproductive consequences of mutations affecting cell division or DNA repair. In these instances, the unrequired sex also suffers the ill-effects of the mutation, posing a serious animal welfare issue. A system to constrain mutations to one sex would therefore be highly beneficial. Here, we overcome these challenges, by creating a bicomponent sex selection system that produces male-only or female-only mouse litters with one hundred percent efficiency, and can also be repurposed to produce postnatal sex-specific phenotypes.

## Results

### An in vitro CRISPR-Cas9 bicomponent system induces *Top1* mutations

We first sought to design a synthetic lethal CRISPR-Cas9 system to generate single-sex litters. For the sgRNA component of our system, we needed to target a gene whose disruption would cause early embryo death, thereby eliminating the chance that mutant offspring would survive to term. In addition, many polytocous species, including mice and domestic pigs, produce an excess of eggs, and only a subset of peri-implantation embryos develop to term^20–22^. Therefore, we aimed to induce embryo loss prior to implantation, in the hope that this would allow a degree of litter size compensation.

**Fig. 1:**
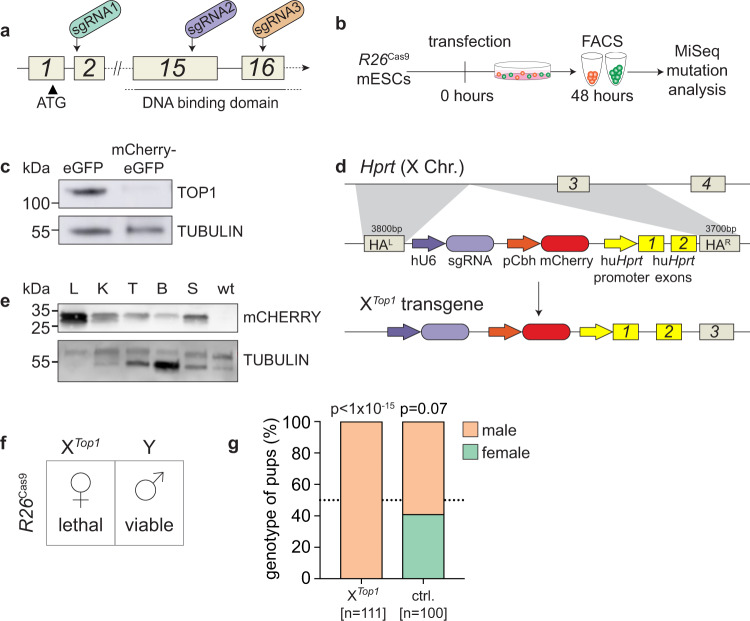
Screening of *Top1* guides and generation of the X^*Top1*^ mouse. **a** *Top1* locus showing sgRNA targeting sites. Exon numbers indicated. **b** Scheme for sgRNA screening. **c** TOP1 western blot in eGFP only and mCherry - eGFP double-positive mESCs after sgRNA2 transfection (TOP1 = 110 kDa, TUBULIN = 50 kDa). The assay was performed twice in two independent experiments. **d** Schematic of X^*Top1*^ transgenic locus. **e** mCherry western blot in X^*Top1*^Y tissues; L liver, K kidney, T testis, B brain, S spleen, wt; C57BL/6 XY liver (TUBULIN = 50 kDa, mCHERRY = 30 kDa). Western blot was repeated three independent times. **f** Mating strategy. **g** Offspring from matings between X^*Top1*^Y males and either homozygous *R26*^Cas9^ females (left column) or control wild-type females (right column). n=number of offspring. *p*-value shows the significance of deviation from 1:1 ratio (Chi-squared test). sgRNA single guide RNA, mESCs mouse embryonic stem cells, FACS fluorescence-activated cell sorting, kDa kilodalton, eGFP enhanced Green Fluorescent Protein, hu human, pCbh hybrid CBA promoter, TOP1 Topoisomerase 1, ctrl control.

We selected the conserved and essential DNA replication and repair factor *Topoisomerase 1* (*Top1*) as our target. In mice, *Top1* disruption causes embryonic lethality at the 4-16 cell stage^23–26^. We cloned guides targeting the *Top1* start codon (sgRNA1) or DNA-binding domain (sgRNA2 and sgRNA3; Fig. 1a) into an mCherry reporter-expressing vector. This vector was then transfected into male mouse embryonic stem cells (mESCs) that constitutively express Cas9 and an eGFP reporter from the autosomal Gt(ROSA)26Sor locus (*R26*^Cas9^; Fig. 1b)^27^. 48 h after transfection, mCherry-eGFP double-positive cells versus eGFP-only control cells were sorted by FACS (Fig. S1A), and the presence of *Top1* mutations was evaluated (Fig. S1B). sgRNA2 had the greatest mutation efficiency, with 52% of observed sequences exhibiting *Top1* mutations, compared with 22% for sgRNA1 and 29% for sgRNA3. The most common sgRNA2-induced mutation was a single nucleotide insertion at the minus 1 position from the Cas9 cut site (−1:1I; Fig. S1C), creating a premature stop codon. Western blotting confirmed loss of TOP1 protein expression in sgRNA2-transfected double-positive mESCs (Fig. 1c).

### Co-inheritance of autosomal sgRNA and Cas9 transgenes induce *Top1* mutations and embryonic lethality

Given its higher mutagenic capacity, we focused on *Top1* sgRNA2 for subsequent in vivo experiments. We first determined whether we could induce lethality in daughters by combining a paternal X-integrated sgRNA transgene with a maternal autosomal Cas9 transgene. We generated an X-integrated sgRNA mouse model using homologous recombination in mESCs, and subsequent generation and breeding of resulting chimeras. The transgene, sgRNA2-mCherry, encoded a U6 promoter-driven sgRNA2, together with a pCbh promoter-driven mCherry reporter. sgRNA2-mCherry was targeted to *Hprt*, which is permissive to integration, and deletion of which is dispensable for fertility^28^ (Fig. 1d). As expected, males carrying X-integrated sgRNA2-mCherry (X^*Top1*^Y) transmitted the transgene only to daughters. mCherry expression was confirmed in X^*Top1*^Y adult tissues by qPCR (Fig. S2A) and western blotting (Fig. 1e).

In order for the paternal X^*Top1*^ transgene to cause lethality in daughters, it would need to be expressed during X^*Top1*^X female embryo development. In female mouse embryos, the paternal X chromosome is silenced from the 4–8 cell stage by imprinted XCI^29,30^. Imprinted XCI is retained in the trophectoderm, but is reversed in the epiblast, after which random XCI ensues^29,30^. In X^*Top1*^X females, the sgRNA2-mCherry transgene was expressed from embryonic day (E) 2.5 and was subsequently subject to both imprinted and random XCI (Fig. S2B, C—see legend for details).

Encouraged by the observation that paternal X-linked sgRNA2-mCherry was detectable in the preimplantation embryo, we wondered whether this transgene would induce lethality in daughters in the presence of maternal Cas9 (Fig. 1f). We mated X^*Top1*^Y males to homozygous autosomal *R26*^Cas9^ females, and genotyped progeny at birth. Strikingly, all pups were male (*n* = 111 pups, *n* = 25 litters, Fig. 1g). Co-inheritance of the X-integrated *Top1* sgRNA and Cas9, therefore, induces female lethality with one hundred percent efficiency.

**Fig. 2:**
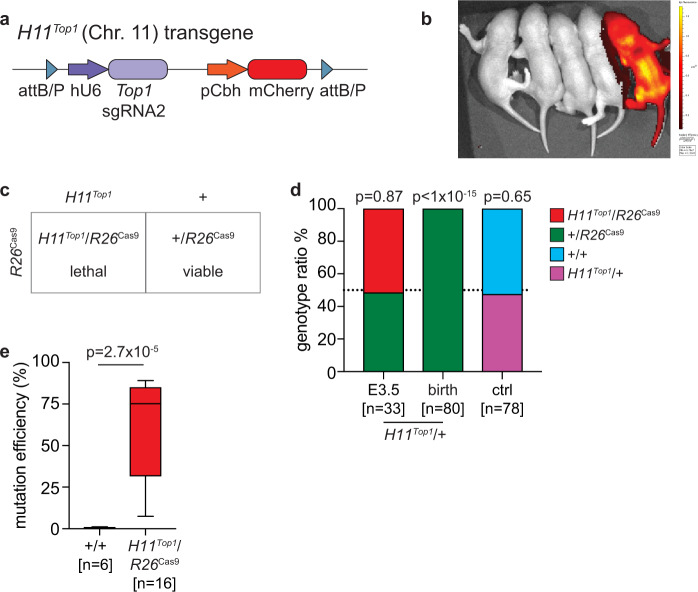
Generating and examining the functionality of the *H11*^*Top1*^ mouse. **a** Schematic of *H11*^*Top1*^ transgenic locus. **b** mCherry in vivo imaging showing *H11*^*Top1*^ mouse (far right). Raw data images provided as a Source Data file. **c** Mating strategy. **d** Offspring from matings between *H11*^*Top1*^/+ males and either homozygous *R26*^Cas9^ females (first two columns) or control wild-type females (right column). *n* = number of offspring. *p*-value shows the significance of deviation from 1:1 ratio (Chi-squared test). **e** *Top1* mutation efficiency in E3.5 wild-type (+/+) and *H11*^*Top1*^/*R26*^Cas9^ embryos (median = 75%). Box plots describe minima, maxima and median with error bars (s.d.). *p*-value; two-tailed Mann–Whitney test, *n* = number of embryos). sgRNA single guide RNA, Top1 Topoisomerase 1, pCbh hybrid CBA promoter, ctrl control, E embryonic day.

In our bicomponent system, the sgRNA2-mCherry transgene was integrated on a sex chromosome and the Cas9 transgene on an autosome. A drawback of this design was that surviving offspring express the Cas9 endonuclease. We, therefore, reversed the bicomponent system, placing sgRNA2-mCherry on an autosome and the Cas9 transgene on the sex chromosome, so that surviving offspring would instead inherit only the sgRNA and thus express no foreign proteins. We inserted the sgRNA2-mCherry transgene into the intergenic autosomal *Hipp11* (*H11*) locus using integrase-mediated recombination in zygotes (Fig. 2a). mCherry expression was confirmed in resulting *H11*^*Top1*^ mice by in vivo imaging of pups (Fig. 2b) and adult tissue qPCR (Fig. S3A). mCherry was detected during development from the blastocyst stage at E3.5 (Fig. S3B).

We tested whether the autosomal sgRNA-mCherry transgene was functional in vivo. We initially bred hemizygous *H11*^*Top1*^/+ males with homozygous autosomal *R26*^Cas9^ females (Fig. 2c), and genotyped progeny at E3.5 and at birth (Fig. 2d). At E3.5, *H11*^*Top1*^/*R26*^Cas9^ and +/*R26*^Cas9^ embryos were recovered in equal proportions (*n* = 33), and *H11*^*Top1*^/*R26*^Cas9^ embryos exhibited *Top1* mutations (Fig. 2e). However, at birth, all pups were +/*R26*^Cas9^ (*n* = 80 pups, *n* = 17 litters). Co-inheritance of *H11*^*Top1*^ and *R26*^Cas9^, therefore, induces embryo lethality with one hundred percent efficiency, confirming that the autosomal sgRNA2-mCherry is functional.

### Generating single-sex litters using sex chromosome-linked Cas9 transgenes

**Fig. 3:**
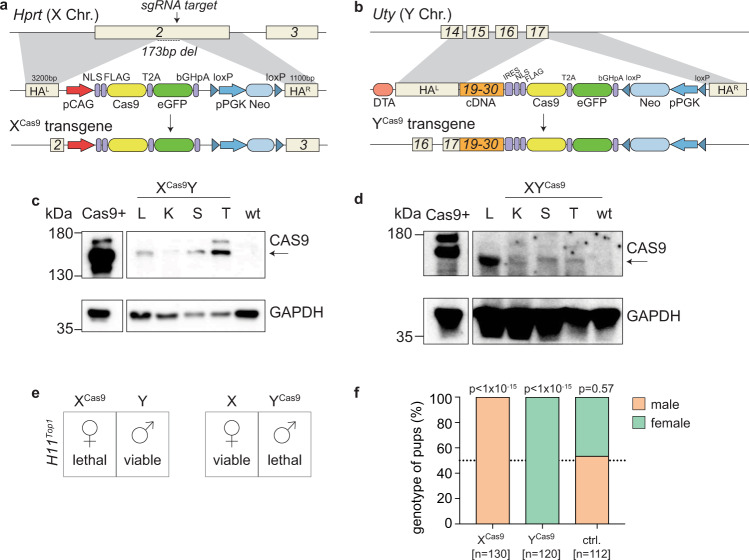
Examining the X^Cas9^ and Y^Cas9^ functionality to generate single-sex litters. **a** Schematic of X-linked Cas9-eGFP transgenic locus. **b** Schematic of the Y-linked Cas9-eGFP transgenic locus. Exon 18 is skipped in wild-type *Uty* expression. Exons 19-30 are inserted into the transgene as cDNA. HA^L^ homology arm left, HA^R^ homology arm right, NLS nuclear localisation signal. **c**, **d** Western blot of X^Cas9^ and Y^Cas9^ tissues. L liver, K kidney, S spleen, T testis. (CAS9 = 158 kDa (arrow), GAPDH = 37 kDa). Western blot has repeated a minimum of three independent times. **e** Mating strategies. **f** Sex genotyping of pups born from X^Cas9^Y males (left) or XY^Cas9^ males (middle) bred with homozygous *H11*^*Top1*^ females or control X^Cas9^Y males (right) mated to wild-type females. *n* = number of pups. *p*-value shows the significance of deviation from 1:1 ratio (Chi-squared test). sgRNA single guide RNA, Chr chromosome, pCAG promoter CMV early enhancer/chicken β-actin, eGFP enhanced Green Fluorescence Protein, pPGK promoter phosphoglycerin kinase, Neo Neomycin, NLS nuclear localisation signal, DTA diphtheria toxin A, wt wild-type, ctrl control.

To constrain embryonic lethality to a specific sex, we used mESC targeting to create two more mouse lines, one expressing Cas9 from the X chromosome (Fig. 3a) and another expressing Cas9 from the Y chromosome (Fig. 3b). In both instances, we used a transgene containing Cas9 linked via a T2A sequence to an eGFP reporter, hereafter termed Cas9-eGFP. A floxed neomycin cassette was also included for targeting selection. For the X chromosome, we used CRISPR-Cas9 to integrate a pCAG promoter-driven Cas9-eGFP into the *Hprt* locus (Fig. S4A, B). For the Y chromosome, we used homologous recombination to integrate Cas9-eGFP in-frame into the ubiquitously expressed *Uty* locus (Fig. S5A). Although *Uty* is not essential for fertility^31^, we confirmed that *Uty* expression was preserved in adult tissues (Fig. S5B).

Males carrying Cas9-eGFP on the X chromosome (X^Cas9^Y) or the Y chromosome (XY^Cas9^) were derived by blastocyst injection and germline transmission from resulting chimeras. As expected, X^Cas9^Y males transmitted Cas9-eGFP only to daughters, while XY^Cas9^ males transmitted Cas9-eGFP only to sons. For the X-integrated Cas9-eGFP, eGFP expression was detected by qPCR in adult tissues (Fig. S4C) and embryos (Fig. S4D). eGFP was also observed by fluorescence microscopy in adult organs (Fig. S4E) and in embryos (Fig. S4F). Western blotting confirmed Cas9 expression in adult tissues (Fig. 3c). Paternal X-linked Cas9-eGFP was expressed from E2.5 in X^Cas9^X embryos, and then subject to both imprinted and random XCI (Fig. S4F, G – see legend for details). For the Y-integrated Cas9-eGFP, eGFP expression was detected by qPCR in adult tissues (Fig. S5C) and in embryos (Fig. S5D) but could not be detected by fluorescence microscopy. However, Cas9 expression was observed in adult tissues by western blotting (Fig. 3d).

We determined whether our *H11*^*Top1*^, X^Cas9^Y and XY^Cas9^ lines could be used to create single-sex litters. We focused initially on male-only litters. We mated X^Cas9^Y males still carrying the neomycin cassette component of the Cas9-eGFP transgene to homozygous *H11*^*Top1*^ females. Strikingly, 102 out of 103 (99%) offspring were male (Fig. S4H). Genotyping and low-pass whole-genome sequencing revealed that the single, exceptional daughter was an XO female (Fig. S4I), a genotype that spontaneously arises at low frequency in laboratory mouse stocks^32^. This female had inherited a maternal X chromosome but no paternal X chromosome, and thus lacked the X-integrated Cas9-eGFP transgene necessary to induce lethality.

We then excised the neomycin cassette from our X- and Y-integrated Cas9-eGFP mouse lines and mated the resulting neo-negative X^Cas9^Y or XY^Cas9^ males with homozygous *H11*^*Top1*^ females (Fig. 3e). Pups derived from X^Cas9^Y matings were exclusively male (*n* = 130 pups, *n* = 36 litters), while those derived from XY^Cas9^ matings were exclusively female (*n* = 120 pups, *n* = 36 litters; Fig. 3f). These mating strategies therefore generated single-sex litters in both directions.

Having shown efficient embryo selection with autosomal, X-linked and Y-linked Cas9 transgenes, we addressed the question of litter size compensation. In total, we had generated four iterations of our bicomponent system in which co-inheritance of Cas9 and *Top1* sgRNA2-induced embryonic lethality (Figs. 1–3). This lethality should result in a 50% reduction in mean litter size. Strikingly, however, for all four crosses studied, the number of offspring was higher than 50%. Matings between X^*Top1*^Y males and *R26*^Cas9^ females (Fig. 1f), *H11*^*Top1*^/+ males and *R26*^Cas9^ females (Fig. 2c), and X^Cas9^Y males and *H11*^*Top1*^ females (Fig. 3e), and XY^Cas9^ males and *H11*^*Top1*^ females (Fig. 3e) produced mean litter sizes that were 66%, 72%, 61% and 61% of controls, respectively (Fig. S6). This finding reveals a significant degree of compensation for embryo loss resulting from the *Top1* sgRNA2 system.

### Sex-linked Cas9 transgenes can generate postnatal sex-specific phenotypes

Finally, we investigated whether our sex-linked Cas9 models could be re-purposed to create sex-specific postnatal phenotypes. As a proof-of-principle, we focused on *Atm*. This DNA repair gene functions in multiple biological processes, including meiosis. However, different meiotic studies necessitate males or females. Male *Atm* mutants are used to study recombination and crossover formation on the mammalian X and Y chromosomes^33–35^. Conversely, female *Atm* mutants are used in studies of the oocyte recombination checkpoint^36,37^. In both cases, the unused sex nevertheless experiences multiple adverse effects, including immunodeficiency, neural defects and thymic lymphomas^38,39^.

**Fig. 4:**
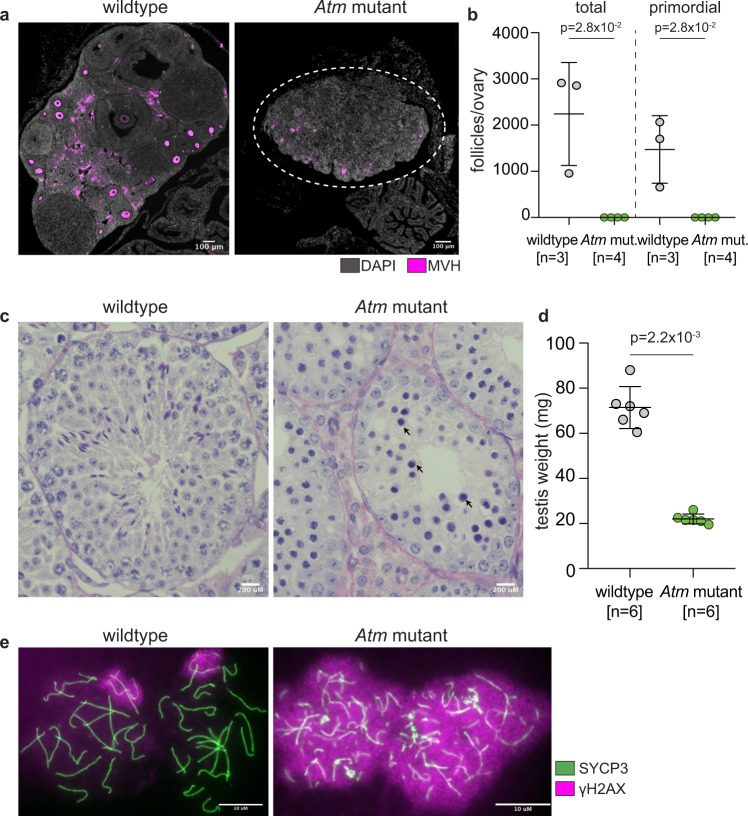
Transient introduction of an sgRNA targeting *Atm* generates a sex-specific phenotype. **a** Ovary sections from wild-type and *Atm* mutant immunostained for germ cell marker MVH (magenta). Dotted circles indicate the degenerated ovary (*n* = 3 wildtype, *n* = 4 *Atm* mutant ovaries). Scale bars: 100 μM. **b** Total and primordial follicle quantification in 8-week-old *Atm* mutant and wild-type ovaries. Error bars: mean ± s.d. Statistical analysis performed using Mann–Whitney test (two-tailed). **c** Periodic-acid Schiff-stained wild-type and *Atm* mutant testis, showing a complete stage IV, midpachytene germ cell arrest (arrows) in the mutant. Scale bars: 200 μM. **d** Testis weights in wild-type (*n* = 6) and *Atm* mutant (*n* = 6) 8-week-old males. Error bars: mean ± s.d. Statistical analysis performed using Mann–Whitney test (two-tailed). **e** Pachytene spermatocytes from wild-type and *Atm* mutant (as in (**d**)) males immunostained for SYCP3 (green) and γH2AX (magenta). Two independent biological replicates. Scale bars: 10 μM. mut mutant.

To constrain the *Atm* mutation to a specific sex, we derived zygotes from matings between wild-type females and either X^Cas9^Y or XY^Cas9^ males, electroporated them with an *Atm* kinase domain-targeting sgRNA, and transferred them into pseudo-pregnant mothers. This is similar to standard methods of embryonic mutagenesis using Cas9 and is expected to produce F0 mosaic mutant offspring. In matings using X^Cas9^Y fathers, *Atm* mutations were induced only in daughters, while in matings using XY^Cas9^ fathers, *Atm* mutations were induced only in sons. Although the *Atm* sgRNA was only transiently present, we could achieve targeting efficiencies equivalent to those expected from zygote electroporation^40^. The mean mutation efficiency was 99.3% (±0.5 s.d.) in *Atm* mutant daughters (*n* = 7) and 94.6% (±12.2 s.d.) in *Atm* mutant sons (*n* = 22). The *Atm* deletion phenotype, assayed within the gonad, was indistinguishable from that observed in existing targeted deletion studies^38,39^, demonstrating that the mosaic mutant offspring are phenotypically equivalent to known homozygous null animals. Female and male *Atm* mutant mice produced no offspring (*n* = 10 mating pairs). At 8 weeks post-partum, ovaries from *Atm* mutant females were atrophic (Fig. 4a) and devoid of oocytes (Fig. 4b). Testes from *Atm* mutant males showed germ cell arrest at mid pachynema, resulting in no sperm in the seminiferous tubules (Fig. 4c) and lower testis weights relative to wild-type males (Fig. 4d). *Atm* mutant chromosome spreads, analysed using immunostaining for axial element marker SYCP3 and DSB-marker γH2AX, exhibited the expected pattern of chromosome fragmentation and persistent DNA damage (Fig. 4e). Thus, in addition to their utility in generating single-sex litters, the X^Cas9^Y or XY^Cas9^ models can be used to create other sex-specific phenotypes.

## Discussion

In conclusion, we show that targeting the essential gene *Top1* in a synthetic lethal system produces single-sex mouse litters. Our system will have immediate benefits for laboratory research, e.g., in the production of genetically modified mice where there is usually a requirement for male blastocysts, or sex-limited research such as the investigation of spermatogenesis. Relatedly, our X- and Y-linked Cas9 transgenes can be used in conjunction with other guide RNAs to generate sex-specific phenotypes, as we demonstrate with *Atm*. This would require that the level of Cas9 expression is sufficiently high in the tissue of interest. Clearly, this is the case in the gonad. In particular, X^Cas9^Y or XY^Cas9^ mice may be crossed to stable sgRNA transgenic lines expressing one or multiple sgRNAs, each transcribed in a ubiquitous, tissue-specific or inducible manner. This greatly facilitates the production of complex multiply gene-deficient animals in order to study epistatic interactions between genes of interest. Such complex mutagenesis is currently laborious in mice, and technically impossible in the case of dominant sterilizing mutations such as Y chromosome gene deficiencies. Although multigene targeting using genomically integrated CRISPR-Cas9 has previously been achieved^15,19,41^, it has not been deployed efficiently in a sex-specific manner. Finally, using genetically loaded Cas9 in pre-implantation embryos is more efficient at generating targeted mutations than provision via injection or electroporation, and allows for better embryo survival^40^.

Given the highly conserved function of *Top1*, our approach could also be amenable to the development and implementation of sex selection in other mammals, e.g. livestock. The advent of CRISPR-Cas9 makes transgenesis in different species relatively straightforward, and safe-harbour loci for transgene integration have been described in pigs, cattle and chickens^42–44^. Furthermore, transgenesis could be achieved by zygote injection rather than by ES cell targeting. Generation and maintenance of these lines would require additional breeding steps, but notably, the transgenes are inert when maintained mono-allelically.

In mice, a benefit of our system is that offspring of the desired genotype are more abundant than would be generated from control breeding. Many species, including mice and domestic pigs, produce more eggs and preimplantation embryos than can be accommodated by the uterus^20–22^. Subsequently, there is a period of litter size adjustment during early development in which excess embryos are lost. The timing of this adjustment period is not well defined and may vary between species. However, a sex-selection system that acts very early in development should allow the greatest opportunity for additional embryos to develop. Presumably, in our system, early elimination of *Top1* mutant embryos permits the development of healthy embryos that would otherwise have been lost during litter size adjustment, thereby maximising the remaining yield of the desired genotype. Pigs exhibit a higher degree of maternal excess ovulation and a longer pre/peri-implantation period than mice^21^. This allows both a greater opportunity for compensation, and a wider time window in which to eliminate embryos of the undesired sex. The level of litter size compensation in pigs may thus potentially be higher than that seen here in mice. In addition, since hormone injections are routinely used to stimulate and synchronise ovulation in livestock^45,46^, there may also be the potential for these stimulation protocols to be adjusted to produce full-size litters of the desired sex.

A further potential area for application is in poultry, where the specific requirement of females in the egg-laying industry results in culling of 6–7 billion male chicks at birth each year worldwide^47^. The sex chromosome complement of birds differs from mammals, with females being heterogametic (ZW) and males, homogametic (ZZ). Since sex is determined by the female gamete, sperm selection is not applicable, and embryo selection is the only remaining option for offspring sex ratio control. For our system to be applicable to poultry, the Cas9 gene must be integrated on the Z or the W chromosome. If Z- or W-derived Cas9 protein is expressed in the oocyte it will be maternally deposited in the zygote. This could potentially trigger lethality even in embryos that do not carry the maternal Cas9-bearing sex chromosome. We envisage that this maternal effect is unlikely to occur, because in poultry the paternal genome does not activate until the late blastocyst stage^48^. Thus, by the time the sgRNA trigger is expressed, maternally derived Cas9 will have been greatly diluted by embryo growth. The issue of maternal Cas9 inheritance could also be entirely removed by choosing a promoter that drives Cas9 expression in the early embryo but not in the oocyte.

In developing any novel gene technology, it is essential to also consider its potential misuses^49^. Here we consider three key questions, namely; the ethics associated with the intentional use of this method, whether this work has any implications for humans, and whether there are any biosafety risks that arise from the work. Most importantly, the requirement for both parents to be transgenically modified in order to select offspring sex means there is no risk of this technology being misapplied for human sex selection. The uses of this technology are to allow sex selection of laboratory animal offspring for research, to enable the generation of sex-limited phenotypes in a laboratory context, and/or to facilitate the development of related methods of offspring sex selection in farmed animals, depending on relevant legislation and consumer acceptability. In all these situations, offspring sex is already controllable by less effective and less humane means—namely culling of unwanted animals, or more rarely by semen selection. Indeed, avoiding the necessity for animal culling is the key driver for this research.

In relation to biosafety, while genomically encoded Cas9 and gRNA can be used to produce a gene drive^50^, we have designed our transgenes to avoid this risk. In our method, the Cas9 and gRNA form an anti-drive in which the undesired gene combination is never passed on at all. Furthermore, the Cas9 lines are not suitable as the basis for a drive system since constitutively expressed Cas9 drives poorly in the female germline and not at all in the male germline^51^. Similarly, our lines with *Top1* gRNA cannot drive even in principle since they are embryo lethal when activated. Our work therefore neither poses any risk of gene drive, nor provides the founding blocks to develop these in the future. There are therefore no dual-use concerns arising from this work.

In summary, we present a 100% effective method for achieving offspring sex ratio selection and/or generation of sex-specific phenotypes in offspring, which is in principle applicable to any species with differentiated sex chromosomes. Debates regarding the use of genetically modified products in agriculture are evolving, with the US Food and Drug Administration (FDA) now approving genetically modified crops and salmon for consumption. The implications of our technology should therefore be widely considered at the ethical and regulatory level.

## Methods

### Maintenance of mouse lines

All mouse lines (*Mus musculus*) were maintained under UK Home Office Regulations, UK Animals (Scientific Procedures) Act 1986, and according to ethical guidelines at the Francis Crick Institute. Permission for animal experiments was granted by The Crick Biological Research Facility Strategic Oversight Committee (BRF-SOC) incorporating Animal Welfare and Ethical Review Body (AWERB) (Project Licence P8ECF28D9). All mice were kept in individually ventilated cages (IVC), with constant access to food, automatic watering systems, and air management systems which control air flow, temperature (20–22 °C) and humidity (49–62 %). The mice were kept under a 12 h light and 12 h dark cycle. The mouse lines were checked daily and maintained in specific pathogen-free (SPF) conditions. Sufficient nesting material and environmental enrichment was provided. X^*Top1*^Y mice were generated on a 129P2/OlaHsd background, in order to take advantage of the GenOway Quick Knock-in approach. After generating a stable line, the X^*Top1*^Y mice were maintained on C57BL/6J background in order to more closely recapitulate the genetic background of the other transgenic lines. The *H11*^*Top1*^ mouse line was generated on a C57BL/6J background and maintained on a C57BL/6J background. X^Cas9^Y and XY^Cas9^ mice were generated and maintained on a C57BL/6 N background. Wild-type control mice were C57BL/6J. 6–10-week-old female and 8–14-week-old male mice were used for matings.

### Embryonic stem cell derivation and maintenance

Embryos were collected at E3.5 by flushing the uterus with Follicle Holding Medium (FHM) from timed mating 6–8-week-old females, and placed in individual wells of a 24-well plate with 500 μl of 2i/LIF. Outgrowths were dissociated and mESCs seeded into a 4-well plate in 2i/LIF. mESCs were passaged by removing 2i/LIF, washing with PBS, followed by dissociation with TrypLE (Gibco), quenching with 2i/LIF and pipetting into a single cell suspension. Following centrifugation at 200 g for 3 min, mESCs were resuspended and seeded in new plates^52^. mESC lines were maintained in 2i/LIF conditions on laminin-coated tissue culture grade plasticware^53^.

### Primer design

All primer pairs used in this study were designed using the publicly available tool Primer3 (http://bioinfo.ut.ee/primer3/). All PCR amplification was carried out using Q5 High-Fidelity DNA polymerase (NEB) at recommended Q5 thermocycling conditions. To amplify *Top1* and *Atm* target exons for MiSeq analysis, primers were designed using Primer3 and extended to contain MiSeq adaptor sequences (see relevant section). All primer sequences are listed in Table S1.

### sgRNA design

All sgRNAs were designed using publicly available in silico tools^54^. Single sgRNAs with a predicted high on-target activity and low off-target activity were selected. Oligonucleotides with BbsI overhangs were annealed and ligated into the relevant vector, according to a published protocol^55^. For transient electroporation of sgRNAs, the sgRNA oligonucleotide was bought from Integrated DNA Technologies. All sgRNA sequences are contained in table S1.

### Fluorescence-activated cell sorting (FACS)

Transfected mESCs were dissociated using TrypLE into a single cell suspension, centrifuged at 200 × *g* for 3 min, and resuspended in sorting media (2% FBS in 2i/LIF). mESCS were filtered (40 μM) and sorted using the Aria Fusion Flow Cytometer with a 100 μM nozzle. mESCS were firstly gated on forward and side scatters properties, followed by gating on either eGFP-mCherry double-positive expression or eGFP only. The eGFP-only population acted as the CRISPR-Cas9 negative control.

### Embryo dissociation and flow cytometry

E9.5–E12.5 embryos were dissociated and prepared for flow cytometry according to a previously published protocol^56^. Dissociated cells were filtered (40 μM) and kept on ice in sterile PBS with 2% FBS prior to flow cytometry and analysis on the MACSQuant VYB. Single cells were analysed on forward and side scatter properties, followed by gating on eGFP or mCherry expression (Table S4.) using FlowJo v10.7 software. Gating strategies for all the embryo samples for either eGFP expression or mCherry expression can be seen in Supplementary Fig. 8 and Supplementary Fig. 9, respectively.

### Generating the X^*Top1*^ mouse line

The *Top1* sgRNA targeting vector was generated using plasmid pX333 as a backbone (Addgene #64073)^57^. The Cas9 cassette was replaced with an mCherry reporter. *Top1* sgRNA2 were inserted using BbsI^55^. The resulting hU6-sgRNA2-pCbh-mCherry construct was used to generate the X^*Top1*^ transgene by GenOway (Lyon, France) using their Quick Knock-in approach in E14Tg2a mESCs derived from 129P2/OlaHsd. Human *Hprt* exons 1 and 2 were inserted into the sgRNA2-mCherry targeting vector. The construct was targeted to the *Hprt* locus in mESCs that lacked exons 1 and 2. Upon recombination of the targeting vector into *Hprt*, gene function was restored. Correctly targeted clones were selected with hypoxanthine-aminopterin-thymidine (HAT) medium. Eight HAT-resistant clones were selected and amplified for confirmation of on-target integration. X- integration clones were confirmed using PCR and Sanger sequencing for validation.

### Generating the *H11*^*Top1*^ mouse line

To generate the *H11*^*Top1*^ targeting vector we repurposed the same hU6-sgRNA2-pCbh-mCherry plasmid used to generate X^*Top1*^. The human U6 promoter-sgRNA cassette and pCbh-mCherry reporter sequence was inserted into the TARGATT MCS plasmid vector #3^58,59^ (Applied Stem Cell). The TARGATT plasmid containing hU6-sgRNA2-pCbh-mCherry was microinjected into attPx3 embryo pronuclei with ϕC31 integrase (sourced from Applied Stem Cell protocols via InsightBio). The plasmid DNA was diluted to 6 ng/μl in 5 mM Tris/0.1 mM EDTA. 10 μl plasmid was mixed with 10 μl TARGATT integrase solution and filtered through a 0.2 μM syringe filter immediately prior to zygotic injection. Embryos were surgically transferred into pseudopregnant females. Founders were screened by in vivo fluorescence imaging at 3-4 days post birth using the IVIS Lumina XR (Caliper LifeSciences) with Living Image 4.4 software, excitation filter at 535 nm and emission filter dsRed. One founder (*n* = 1/25) was mCherry positive and germline transmitted.

### Generating the X^Cas9^ mouse line

The Cas9-eGFP targeting vector was generated using the pX330 (Addgene #42230)^60^ plasmid backbone, containing a pCAG driven 3X FLAG-NLS-Cas9-T2A-eGFP construct. X chromosome homology arms, amplified from C57BL/6J DNA, and a LoxP-flanked pPGK-Neomycin cassette were inserted using directional cloning or Gibson Assembly (NEBuilder HiFi DNA Assembly Cloning Kit). C57BL/6N mESCs were maintained in serum/LIF conditions and transfected with the Cas9-eGFP targeting vector plasmid and an sgRNA targeting *Hprt* exon 2 using Lipofectamine 2000, according to manufacturer’s instructions. Targeted mESC clones were selected by G418 (270 mg/ml) for 8–10 days. Forty-eight surviving clones were picked into a 96-well plate and expanded. PCR genotyping was performed on extracted DNA in a total volume of 25 μl (12.5 μl NEB Q5 High-Fidelity Master Mix, 10 mM each primer), using primer forward and reverse pairs aligning to the endogenous *Hprt* locus and to the transgene construct. Resultant PCR amplicons were analysed by gel electrophoresis for corresponding to the expected amplicon size, and by Sanger sequencing. Of 48 clones, 9 were found to be successfully targeted (19%). Targeted mESC clones were injected into albino C57BL/6J-Tyr<c-Brd> blastocysts and surgically transferred into pseudopregnant females. X^Cas9^Y mESC contribution to founders was assessed by coat colour. High contribution transgenic males were bred with C57BL/6J-Tyr<c-Brd> albino females, and offspring with black coat colour were genotyped for the transgene to confirm germline transmission.

### Generating the Y^Cas9^ mouse line

In order to generate XY^Cas9^, we repurposed the Cas9-eGFP plasmid vector used to generate the X^Cas9^Y mouse model. The Cas9-eGFP plasmid contained a pCAG driven 3X FLAG-NLS-Cas9-T2A-eGFP construct. The Cas9-eGFP cassette was targeted to the Y-linked *Uty* locus, which is ubiquitously expressed. In order to maintain *Uty* expression, the Cas9-eGFP plasmid was targeted to *Uty* exon 17, which is retained in all *Uty* isoforms. A plasmid vector containing exons 19-30 as cDNA (exon 18 is excised during normal *Uty* transcription) was inserted into the plasmid vector upstream of an IRES cassette, Following the IRES was Cas9-eGFP. The plasmid was electroporated into C57BL/6N mESCs following a proprietary protocol carried out by GenOway (Lyon, France). Clones underwent positive neomycin selection and negative DTA selection. 24 positive clones were selected by a pre-screen and five were confirmed positive Y-integration clones by PCR and Sanger sequencing. Following this result, GenOway also performed sequencing analysis spanning the Y-integration region, and concluded that four positive clones were correctly targeted.

### Genotyping

Pups were genotyped using assays for the Cas9-eGFP transgene, the mCherry transgene, and sexed by the presence of Y-linked gene *Sry* using Transnetyx assays. X^Cas9^ hemizygous versus homozygous females were distinguished by genotyping for the *Hprt* exon 2 deletion (primer sequences available in Table S1.). Thermocycling conditions as follows: 95 °C (3 min), 30 X cycles of 95 °C (20 s), 60 °C (15 s), 72 °C (20 s) followed by 72 °C (3 min) and 4 °C (hold). The XO female generated from breeding X^Cas9^Y males with *H11*^*Top1*^ homozygous females, was characterised by DNA extraction from ear biopsy tissue (see relevant methods); X-chromosome and transgene copy number analyses using digital droplet PCR (see relevant methods), and low-pass whole genome Oxford Nanopore Technologies sequencing (see relevant section). *H11*^*Top1*^ hemizygous versus homozygous mice were distinguished using mCherry copy number analysis using Transnetyx raw data.

### MiSeq high throughput sequencing and indel analysis

Lysis was performed using lysis buffer (10X KT buffer, 10% NP40) with proteinase K (1 mg/ml) digestion. Target *Top1* exons were PCR-amplified using MiSeq PCR primers (Table S1.) in a total volume of 25 μl (12.5 μl NEB Q5 High-Fidelity Master Mix, 5 mM each primer) Correct PCR amplification was confirmed by gel electrophoresis. Resultant PCR amplicons were purified using solid-phase reversible immobilisation (SPRI) beads (according to manufacturer’s protocol)^61^ and resuspended in 15 μl nuclease-free water. To prepare libraries, 1 μl DNA was PCR amplified with NXT Primer Mix (IDT-8nt) using Q5 High-Fidelity Master Mix (NEB). Thermocycling conditions as follows: 95 °C (3 min), 10 X cycles of 95 °C (30 s), 55 °C (15 s), 72 °C (30 s) followed by 72 °C (5 min) and 12 °C (hold). PCR products were purified using SPRI bead clean. Libraries were quality controlled using Glomax (Promega, manufacturer’s procedure) and pooled. Pooled libraries were quantified using Qubit Lifetech and HSD1000 Tapestation Agilent. MiSeq libraries were sequenced on Illumina MiSeq-Nano 250PE to generate paired-end (2 × 250 bp) sequencing reads. Resultant reads were demultiplexed and fastq files were collapsed using FastX Toolkit (v0.0.13) [https://github.com/agordon/fastx_toolkit]. To assess the rate of indel-production by CRISPR-Cas9, the reads were aligned to the mouse reference genome mm10 with the Burrows-Wheeler Alignment tool (BWA, v0.7.170)^62^ using the *mem* algorithm with default settings and then analysed using the R package CrispRVariants (v1.14.0)^63^. Scripts are deposited on github [https://github.com/jzohren/crispr-miseq]. Raw MiSeq read counts are available in the associated Source Data files. In each experiment, SNV; single nucleotide variant, I; insertion, D; deletion. The plus/minus number refers to the position that the mutation has occurred, relative to three nucleotides upstream of the PAM. The second number refers to the number of nucleotides that have been inserted or deleted. Data will be made available upon reasonable request.

### Low-pass whole genome Oxford Nanopore Technologies sequencing

DNA was extracted using the phenol-chloroform method as described previously^64^. DNA libraries were prepared in accordance with the Oxford Nanopore Technologies (ONT) SQK-LSK109 Ligation Sequencing protocol, with multiplexing using the EXP-PBC096 kit. Libraries were sequenced on a FLO-MIN106D R9.4.1. flow cell on the MinION MIN-106B. Basecalling was performed using ONT-Guppy v3.2, and data was mapped using minimap2 v2.17^65^ and SAMtools v1.9^66^, and analysed using base R v4.0.2 and Excel. Raw chromosome read counts are available as Supplementary Table 5 and also available as Source Data. Data will be made available upon reasonable request.

### Quantitative PCR analysis

RNA was extracted using TRI Reagent (Sigma-Aldrich), according to the manufacturer’s protocol. cDNA was synthesised using the Thermo Scientific First Strand cDNA Synthesis Kit, according to the manufacturer’s protocol. Samples were analysed in triplicate, in 10 μl total volume (5 μl TaqMan 2X Universal PCR Master Mix, 0.5 μl TaqMan probe, 2.5 μl nuclease-free water, 2μl cDNA). Thermocycling conditions as follows: 95 °C (10 min), 40 X cycles of 95 °C (15 s), 60 °C (1 min), followed 4 °C (hold). Resulting ddCt values were calculated by normalising to *Gapdh* expression from C57BL/6 samples unless otherwise described. TaqMan probes used are available in Table S2.

### Digital droplet qPCR

DNA was extracted by phenol-chloroform precipitation. Digital droplet qPCR (ddPCR) reactions were performed in 20 μl total volume with 20 ng DNA, according to manufacturer’s instructions (Bio Rad ddPCR Supermix for Probes). The ddPCR was performed on a Bio-Rad PCR machine and analysed using QuantaSoft v1.7.4.0917. ddPCR was performed using Taqman copy number probes and are available in table S2. Thermocycling conditions as follows: 95 °C (10 min), 39 X cycles of 94 °C (30 s), 60 °C (1 min), followed by 98 °C (10 min) and 4 °C (hold).

### Protein extraction and western blot

Protein was extracted from samples using 1X RIPA buffer with additional phosphatase and protease inhibitors, and PMSF. Upon adding protein extraction buffer to samples, samples were kept on ice for 30 m, the following centrifugation at 5900 × *g* at 4 °C for 10 m. The supernatant was collected and protein quantified using a bicinchoninic acid (BCA) assay and analysed using Kaleido 2.0. Proteins were separated using PAGE and transferred to 0.45 μm pore Nitrocellulose membrane (Amersham Protran). Membranes were blocked with 5% skimmed milk/TBST for 1 h at room temperature and incubated with primary antibodies overnight at 4 ^o^C. CAS9 (Novus Bio) and TOP1 (Abcam) antibodies were used at 1:500, α-TUBULIN (Sigma) at 1:2000, GAPDH (Santa Cruz Biotechnology) at 1:3000 dilutions. Secondary antibodies conjugated to HRP were used (anti-mouse IgG-HRP (Santa Cruz, 1:4000 dilution), or anti-rabbit IgG-HRP (Cell Signalling 1:4000 dilution)) and signals were detected using Clarity Western ECL Substrate (Bio-Rad). Antibodies used are available in table S3. Raw blots are available as Source Data.

### Southern blot

DNA was extracted by phenol-chloroform precipitation, digested using appropriate restriction enzymes, and phenol-chloroform precipitation repeated. DNA was loaded onto a 1% agarose gel and gel electrophoresis run overnight at 29 V, followed by the addition of bromophenol blue, and further running at 50 V for 2–3 h. Following gel electrophoresis, the agarose gel was treated by washing in depurination (0.25 M HCl), denaturation (1.5 M NaCl, 0.5 M NaOH) and neutralisation (1.5 M NaCl, 0.5 M Tris pH 7.5) buffers and overnight blotting onto a positively charged nylon membrane. After blotting, the DNA was fixed by UV crosslinking (1200U joules, 2 m) and drying. The membrane then underwent hybridisation to the Neomycin probe (Fig. S7, Table S1), produced according to manufacturer’s instructions (Roche DIG probe synthesis kit) and incubation overnight in a hybridisation oven at the optimal temperature (48 °C for Neomycin). Post-hybridisation, the membrane was washed (2X SSC, 0.1% SDS) at room temperature, and at 65 °C (0.1X SSC, 0.1% SDS). Following this, the membrane was blocked with blocking buffer and incubated for 30 m at room temperature with anti-DIG antibody (1:40,000; Roche DIG Luminescent Detection Kit; Table S3.), washed (maleic acid, 0.3% tween-20), and exposed to CSPD in detection buffer under darkness before film development. Raw blots are available as Source Data.

### Embryo electroporations and post-natal *Atm* mutation analysis

Zygote stage embryos were generated by in vitro fertilisation with either X^Cas9^Y or XY^Cas9^ males and wild-type females. Zygotes were washed through droplets of opti-MEM prior to electroporation with Nepagene 21 (5 mm). sgRNAs were prepared to 12 μM final concentration and brought to room temperature for 10 mins immediately prior to addition to electroporation mix. Fifty microliters of electroporation/RNA mix was added into the electroporation chamber and impedance measured to be at 0.48–0.52 kOhm. Embryos were transferred into pseudo-pregnant methods and left to litter down. At 2 weeks, pups were ear biopsied, DNA extracted, and MiSeq PCRs performed (see relevant methods sections). Daughters from X^Cas9^Y fathers showed mutation efficiencies of 99.7, 99.4, 98.4, 98.8, 99.3, 99.7, and 99.8%. Non-mutant brothers showed a mean mutation efficiency of 0.18% ± 0.19 s.d (*n* = 24 males). Sons from XY^Cas9^ fathers showed mutation efficiencies of: 99.8, 99.6, 78.3 and 51.9% (electroporation round 1), and 99.9, 99.9, 99.9, 99.9, 99.9, 85.3, 99.9, 99.9, 92.1, 73.7, 99.9, 99.9, 99.9, 99.9, 100, 99.9, 99.9 and 99.9% (electroporation round 2). Non-mutant sisters showed a mean mutation efficiency of 0.04% ± 0.03 s.d (electroporation round 1, *n* = 5 females) and 0.03% ± 0.02 s.d (electroporation round 2, *n* = 20 females).

### Ovary section and immunofluorescence

Ovaries were collected at post-natal week 8 for wild-type and *Atm* wild-type females and fixed in 4% paraformaldehyde (PFA) overnight at room temperature. Fixed ovaries were washed with phosphate-buffered saline (PBS), embedded in paraffin and sectioned at 6  μM. For immunofluorescence, slides were deparaffinised and rehydrated by a series of xylene and ethanol, prior to antigen retrieval (0.1 M sodium citrate, 30 min). Sections were blocked for 15 min (5% bovine serum albumin, BSA, in PBS/Tween 20), and incubated with a germ cell marker primary antibody at room temperature for 1 h (rabbit anti-MVH; 1:100; antibodies listed in Table S3), followed by incubation with a secondary antibody (Alexa Fluor® 594, dilution 1:200) at room temperature for 1 h. Slides were mounted with Vectashield plus DAPI (Thermo-Fisher). Images were obtained using Olympus upright BX63 microscope with associated robotic slide loader with an Excite Exacte Measured Metal halide source and built-in Koehler illumination for transmitted light. Captured images were analysed using QuPath v0.2.3 (Open Source).

### Follicle quantification

Per mouse, one ovary and every sixth section per ovary was counted for the presence of primordial, primary, secondary and antral follicles (considered as total in Fig. 4).

### Immunofluorescence of testis nuclear spreads

Testis were collected at post-natal week 8 for wild-type and *Atm* mutant males. Glass slides (ThermoFisher AA00008032E00MNT10) were boiled in water for 10 min and dried completely. Testes were dissociated in Roswell Park Memorial Institute (RPMI) medium. 100 μl of cell suspension was transferred to each slide, with the addition of 50 μl 0.05% TritonX-100 (in water) and kept at room temperature for 10 min. Cells were then fixed in 2% PFA/0.02% sodium dodecyl sulfate (SDS) (in PBS) at room temperature for 1 h, washed with water, and dried. Slides were blocked (0.15% BSA/0.1% Tween20 in PBS) at room temperature for 1 h. Slides were incubated with primary antibodies (SYCP3 1:100 and γH2AX 1:250; antibodies listed in Table S3) in a humidified chamber at 37 ^o^C overnight. Secondary antibodies (Alexa Fluor® 488 and Alexa Fluor 568) were applied in blocking buffer at 37 ^o^C for 1 h. Samples were then washed in PBS at room temperature and mounted in Vectashield with DAPI. Images were taken using Deltavision Microscopy System (100x/1.35NA Olympus UPlanApo objective; GE Healthcare).

### Testis histology

Testis were collected at post-natal week 8 for wild-type and *Atm* mutant males and fixed overnight in Bouin’s solution. Fixed samples were washed in 70% ethanol, embedded in paraffin, sectioned, and stained with Periodic Acid-Schiff staining. Images were taken using Olympus BH2 microscope with 40x/0.70NA Olympus SPlan objectives.

### Litter size quantification

Every experimental and control mating female pregnancy was recorded and females were checked for pups that had been born. Pups that survived to weaning stage were ear biopsied for genotyping at ~14 days post birth and counted for litter size measurements. Pups that died prior to weaning were genotyped if tissue was available. Pregnancies that produced pups that died shortly after birth but tissue could not be recovered (e.g., cannibalisation) were counted but not included in total litter size: X^*Top1*^Y x *R26*-Cas9 = 0, X^*Top1*^Y x Wildtype = 1, *H11*^*Top1*^ x *R26*-Cas9 = 0, *H11*^*Top1*^ x Wildtype = 0, X^Cas9^Y x *H11*^*Top1*^ = 3, X^Cas9^Y x Wildtype = 2, XY^Cas9^ x *H11*^*Top1*^ = 0, XY^Cas9^ x Wildtype = 0. Total litter losses (no pups found after a successful pregnancy) were counted: X^*Top1*^Y x *R26*-Cas9 = 2, X^*Top1*^Y x Wildtype = 2, *H11*^*Top1*^ x *R26*-Cas9 = 0, *H11*^*Top1*^ x Wildtype = 1, X^Cas9^Y x *H11Top1* = 1, X^Cas9^Y x Wildtype = 3, XY^Cas9^ x *H11*^*Top1*^ = 3, XY^Cas9^ x Wildtype = 1.

### Statistical analysis

To determine if the number of male or female pups born from experimental matings deviated from an expected 50:50 Mendelian frequency a Chi-squared test was performed. Expected was considered an equal 50:50 male:female ratio, and Observed was the quantified male and female number. Sample size was considered to be sufficient number of pups to accurately make the assumption that single-sex litters could not be down to random chance. To determine if mutation efficiency in mCherry-eGFP samples deviated from negative control eGFP only samples, a Mann–Whitney test for non-parametric data was performed. To determine the difference in average litter size between experimental and control samples, a Mann–Whitney test for non-parametric data was performed.

### Reporting summary

Further information on research design is available in the Nature Research Reporting Summary linked to this article.

## Supplementary information

Supplementary Information

Reporting Summary

## Data Availability

The data that support the findings of this study are available from the corresponding author upon reasonable request. MiSeq and low-pass Whole-Genome Sequencing read counts are available in the Source Data file. Mouse reference genome mm10 was used for mapping the reads in this study. Source data are provided with this paper.

## Source data

Source Data

## Code availability

Code used to analyse the MiSeq CRISPR-Cas9 experiments throughout the manuscript has been deposited on GitHub [https://github.com/jzohren/crispr-miseq]. The DOI for the GitHub repository is: 10.5291/zenodo.5557228. Any remaining code that supports the findings of this study is available from the corresponding author upon reasonable request.
