# Supplementary material for: CRISPR-Cas9 effectors facilitate generation of single-sex litters and sex-specific phenotypes
Source: Nat Commun. 2021 Dec 3;12:6926. doi: 10.1038/s41467-021-27227-2 (PMC8642469; doi:10.1038/s41467-021-27227-2)
Supplement: Supplementary file 1 — Supplementary Information [file 41467_2021_27227_MOESM1_ESM.pdf]

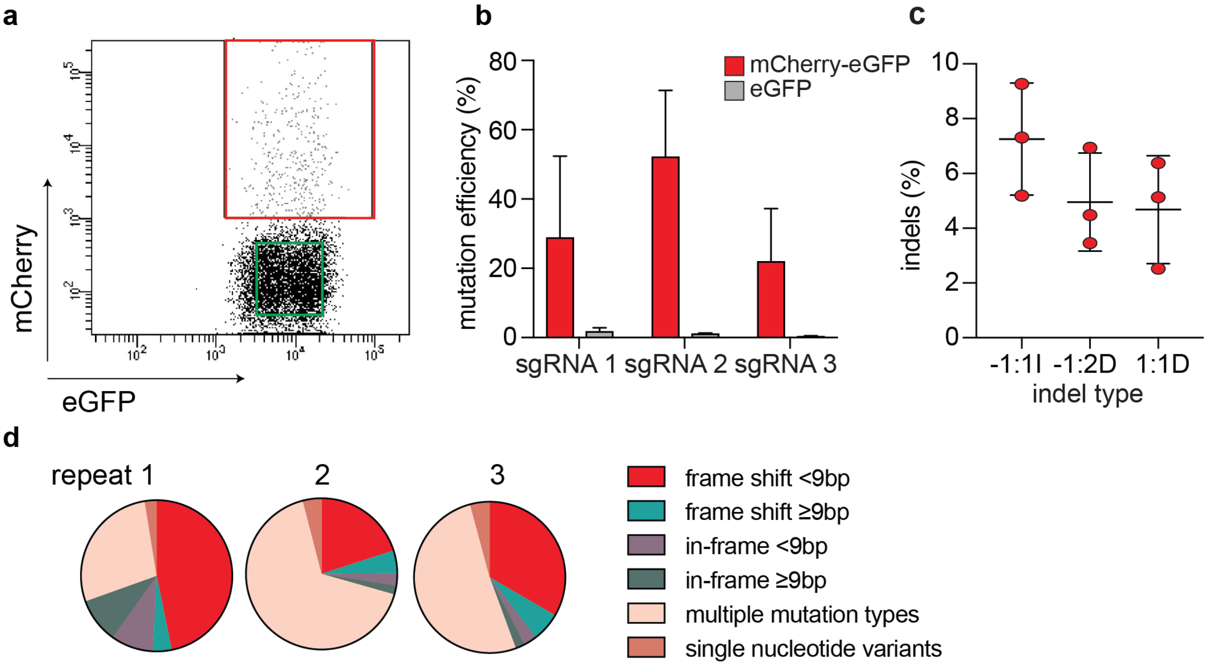

Supp Fig. 1 Screening of *Top1* guides. (**A**) Representative example FACS plot of mCherry-eGFP double-positive mESCs (red border) and eGFP single-positive mESCS (green border). (**B**) Quantification of mutation efficiency. Error bars: mean + s.d. (n=3 replicates of 3 independent experiments). (**C**) Occurrence of *Top1* sgRNA2 mutation types. D, deletion; I; insertion. Error bars: s.d. (n=3 replicates of 3 independent experiments) (**D**) Spectrum of *Top1* sgRNA2 mutation types in each of the three replicates of mCherry-eGFP mESCs. Multiple mutation types refers to reads that contain more than one indel mutation. Abbreviations; sgRNA; single guide RNA, indel; insertion or deletion, eGFP; enhanced green fluorescent protein.

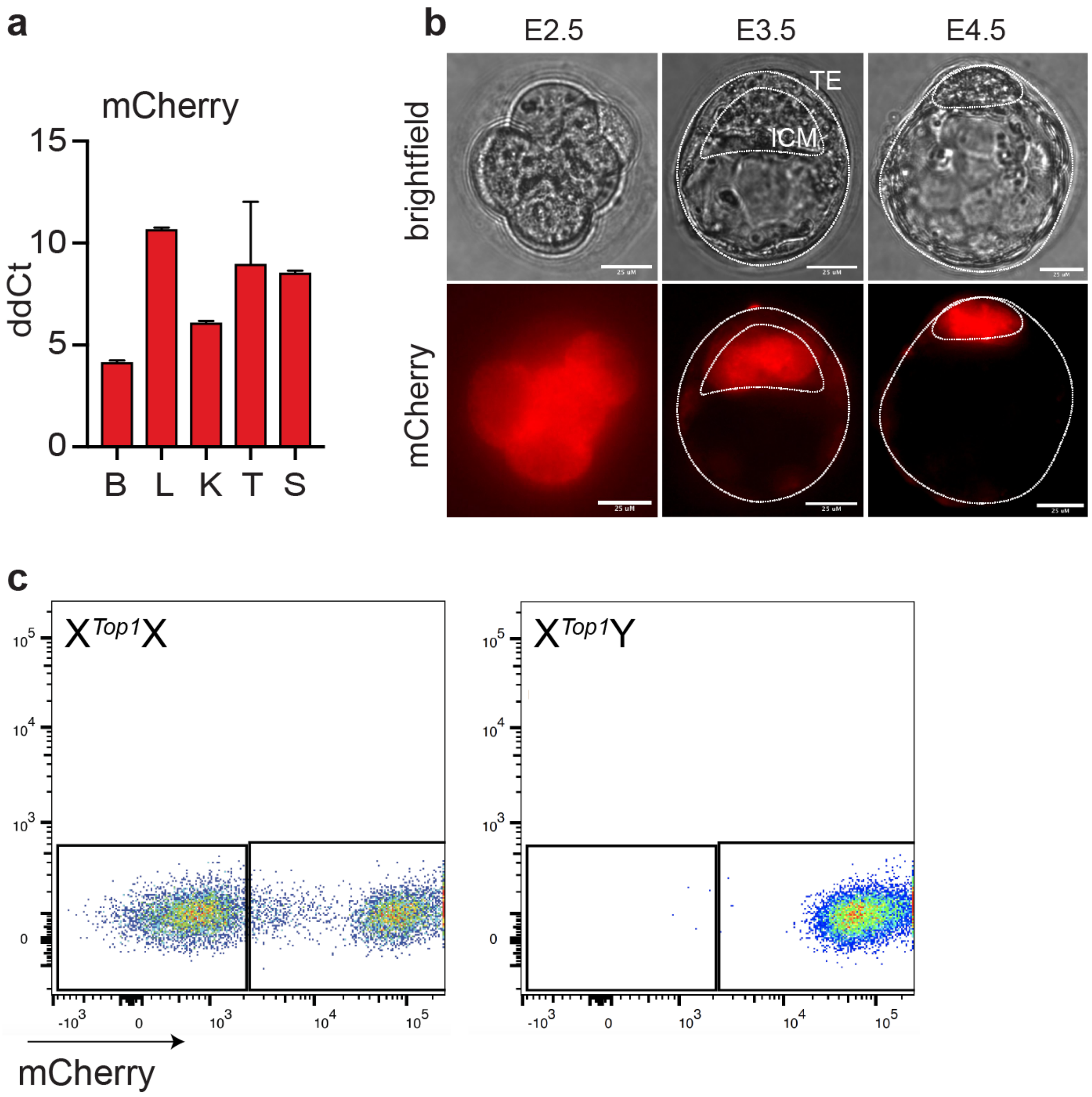

Supp Fig. 2 Characterising the X^{Top1} mouse model. (**A**) mCherry qPCR. Adult tissue Ct values normalised to *Gapdh* Ct from wildtype adult liver sample. B; brain, L; liver, K; kidney, T; testis, S; spleen. Error bars: mean + s.d. (n=3 replicates). (**B**) Fluorescence microscopy of X^{Top1}X embryos derived from X^{Top1}Y fathers mated to wild type mothers, demonstrating that the sgRNA2-mCherry transgene is subject to imprinted XCI. At E2.5 mCherry expression is observed. At E3.5 expression is reduced in the trophectoderm (TE), where imprinted XCI is sustained, but is higher in cells of the inner cell mass (ICM), where X-chromosome reactivation takes place. mCherry expression remains low in the trophectoderm but persists in presumptive epiblast at E4.5. Scale bar= 25 μM. n=27 embryos. (**C**) FACS analysis of E9.5-E10.5 X^{Top1}X embryos derived from X^{Top1}X mothers

mated to wild type fathers. If the sgRNA2-mCherry transgene escaped XCI, all cells would express it. However, this is not the case: in $X^{Top1}X$ embryos (n=5), 62% of cells are mCherry-positive and 38% mCherry-negative. The sgRNA2-mCherry transgene is therefore susceptible to random XCI. In control $X^{Top1}Y$ males (n=4), 99.9% of cells are mCherry positive See Supp. Fig 9 for gating strategy. Abbreviations; E; Embryonic day.

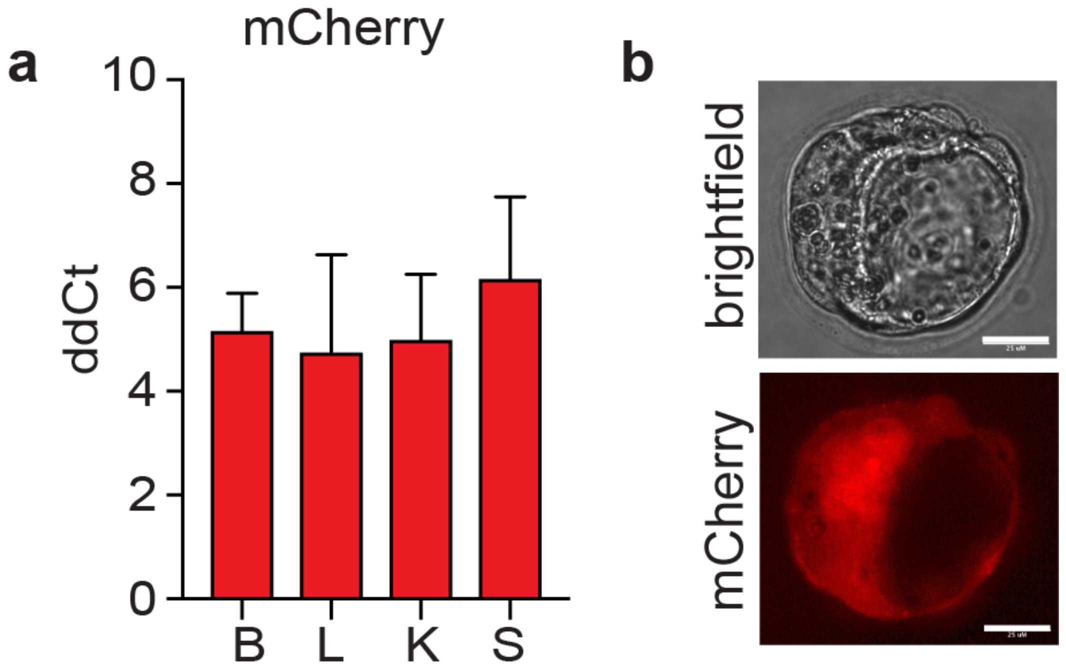

Supp Fig. 3. Characterising *H11*^{Top1} mouse model. (**A**) mCherry qPCR. Adult tissue Ct values normalised to *Gapdh* Ct from a wildtype adult liver sample. B; brain, L; liver, K; kidney, S; spleen. Error bars: mean + s.d. (n=3 biological replicates and n=3 technical replicates). (**B**) Fluorescence imaging of a *H11*^{Top1} E3.5 blastocyst (n=16 embryos analysed). Scale bar: 25μM.

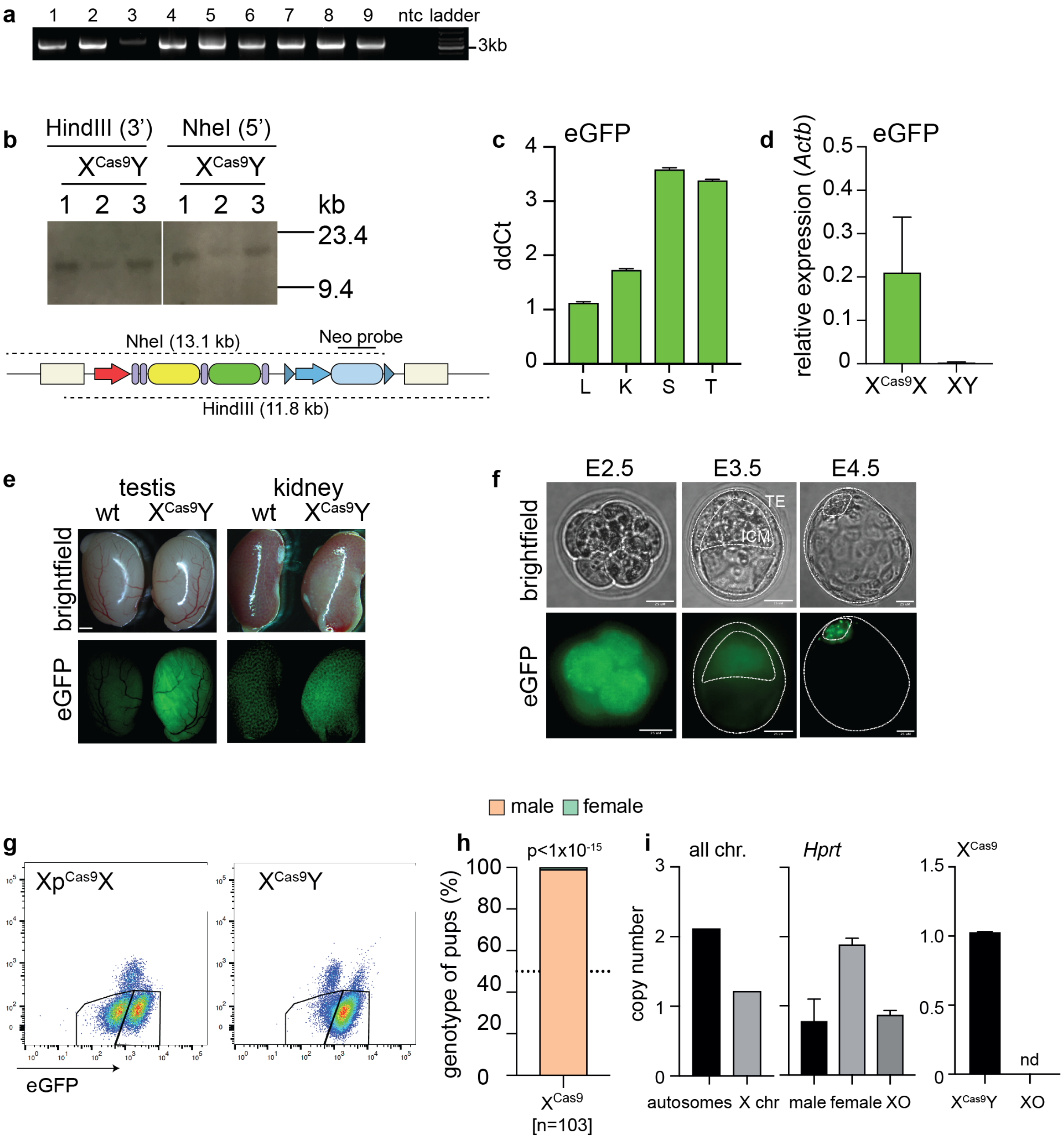

Supp Fig. 4. Characterising $X^{Cas9}Y$ mouse model. (**A**) PCR genotyping gel electrophoresis of nine on-target insertion mESC clones. ntc; no template control. Expected size: 3.5 kb. PCR analysis was performed a minimum of three times. (**B**) Southern blot of X^{Cas9} transgene. Expected size: HindIII 11.8kb, NheI 13.1kb. (**C**) qPCR of eGFP expression in $X^{Cas9}Y$ adult tissues, normalised to *Gapdh* Ct values in wildtype adult liver sample. L; liver, K; kidney, S; spleen, T; testis. Error bars: mean values + s.d. (n=3 technical replicates). Southern blot was performed a minimum of three times on three independent biological samples. (**D**) qPCR of eGFP expression from $X^{Cas9}X$ (n=5) and XY E3.5 (n=4) independent

littermate embryos. Ct values normalised to *Actb* Ct from XY^{Cas9} adult liver tissue (same control as Supplementary Fig. 5C). Relative expression; 2^{-ddCt}.. Error bars: mean values +s.d. (**E**) Fluorescence microscopy of $X^{Cas9}Y$ adult tissues. Two independent animals were analysed. (**F**) Fluorescence microscopy of $X^{Cas9}X$ embryos derived from $X^{Cas9}Y$ fathers mated to wild type mothers, demonstrating that the Cas9-eGFP transgene is subject to imprinted XCI. At E2.5 (n=22) eGFP expression is observed. At E3.5 (n=9) eGFP expression is reduced in the trophectoderm (TE), but high in cells of the inner cell mass (ICM). At E4.5, (n=5) eGFP expression remains low in the trophectoderm at E4.5 but higher in the presumptive epiblast. Scale bar= 25 μM. (**G**) FACS analysis of E11.5 $X^{Cas9}X$ embryos derived from $X^{Cas9}Y$ fathers mated to wild type mothers, demonstrating that the Cas9-eGFP transgene is susceptible to random XCI. In $X^{Cas9}X$ embryos (n=9), 47.3% of cells are eGFP-positive and 52.7% eGFP-negative. In control $X^{Cas9}Y$ males (n=2), 86% of cells are eGFP-positive. See Supp. Fig 8 for gating strategy. (**H**) Sex ratio of pups born from $X^{Cas9}Y$ males bred with *H11*^{Top1} homozygous females. Statistical analysis: Chi-squared test to evaluate deviation from expected Mendelian 50:50 ratio. (**I**) Low-pass whole genome sequencing (all chromosomes; supplementary table 5) and ddPCR (*Hprt,* X^{Cas9}). ddPCR samples normalised to *Tfrc* in wildtype ear biopsy DNA. nd; not detected. Error bars: s.d. (n=3 replicates). Abbreviations; ntc; no template control, eGFP; enhanced Green Fluorescent Protein, wt; wildtype, E; embryonic day, chr; chromosome, nd; not detected.

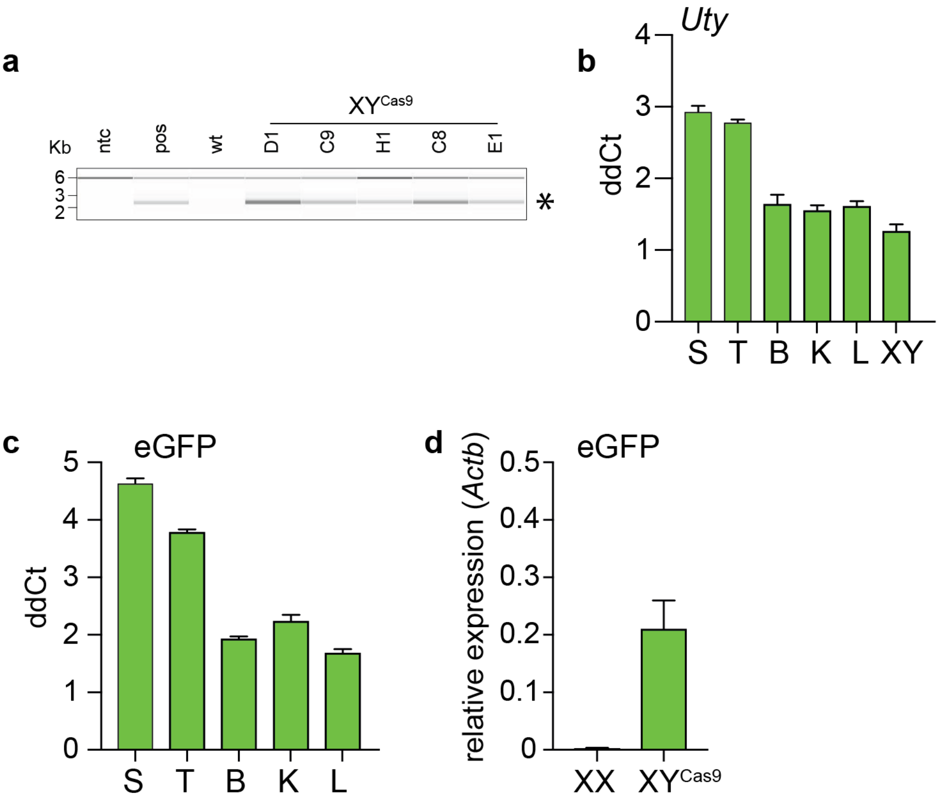

Supp Fig. 5. **Characterising the XY^{Cas9} mouse model.** (**A**) PCR genotyping gel electrophoresis of five Y^{Cas9} on-target insertion mESC clones. ntc; no template control, pos; positive control targeting vector, wt; wildtype. Expected size: 2.8 kb (asterisk). (**B, C**) qPCR of *Uty* and eGFP expression in XY^{Cas9} adult tissues, normalised to *Gapdh* Ct value in adult wildtype liver sample. L; liver, K; kidney, S; spleen, T; testis. XY; male liver sample for normal *Uty* expression. Error bars: mean + s.d. (n=3 replicates). (**D**) qPCR of eGFP expression in XX (n=8) and XY ^{Cas9} (n=12) littermate E3.5 embryos, normalised to *Actb* Ct from adult liver XY^{Cas9} (same control sample as Supplementary Fig. 4D). Relative expression; 2^{-ddCt}. Error bars: mean + s.d. Abbreviations; ntc; no template control, pos; positive control, wt; wildtype, eGFP; enhanced green fluorescent protein.

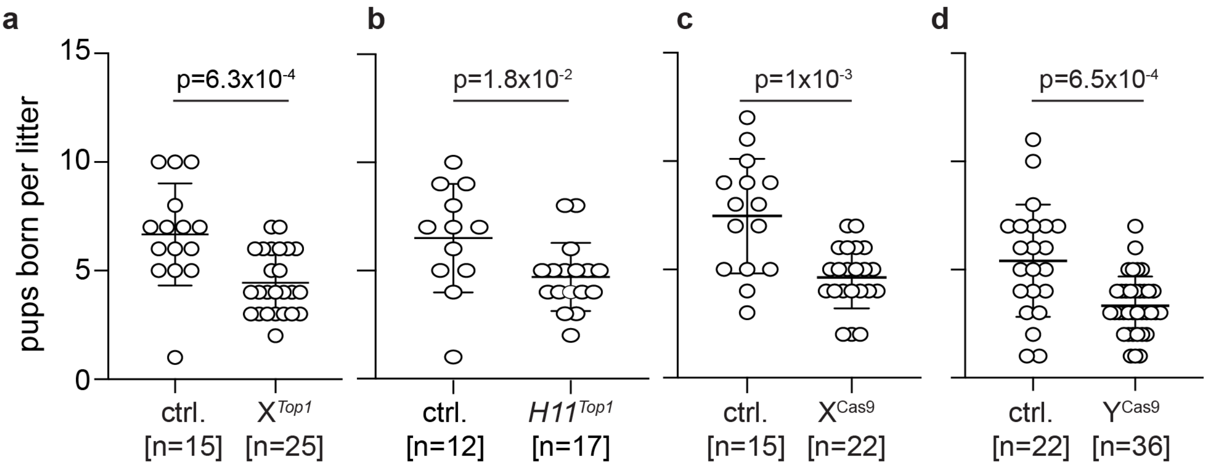

Supp Fig. 6. **Effects of synthetic lethal system on litter size. (A)** Pups born per litter from X*^Top1^*Y males bred with control wildtype females (left) or *R26*^{Cas9} females (right). Mean: ctrl=6.7, X^{Top1}=4.4. Statistically significant increase in mean litter size compared to expected litter size of 50% of control (p = 0.02; Mann-Whitney). (**B**) Pups born per litter from *H11*^{Top1} males bred with control wildtype females (left) or *R26*^{Cas9} females (right). Mean: ctrl=6.5, *H11*^{Top1}=4.7. Statistically significant increase in mean litter size compared to expected litter size of 50% of control (p = 0.01; Mann-Whitney) (**C**) Pups born per litter from X^{Cas9}Y males bred with wildtype females (left) or homozygous *H11*^{Top1} females (right). Mean: ctrl=7.5, X^{Cas9}=4.6. (**D**) Pups born per litter from XY^{Cas9} males bred with wildtype females (left) or homozygous *H11*^{Top1} females (right). Mean: ctrl=5.4, Y^{Cas9}=3.3. Increase in mean litter size compared to expected 50% of control (X^{Cas9}; p=0.07, Y^{Cas9} ; p=0.1, Mann-Whitney). (**A-D**) n=number of litters. p-value shown=significant change in observed mean litter size (Mann-Whitney test, two-tailed). Error bars = mean ± s.d. Abbreviations; ctrl; control.

cagccaatatgggatcggccattgaacaagatggattgcacgcaggttctccggccgcttgggtggagaggctattcggctatgactgggcac
aacagacaatcggctgctctgatgccgccgtgttccggctgtcagcgcaggggcgcccggttctttttgtcaagaccgacctgtccggtgccctg
aatgaactgcaggacgaggcagcgcggctatcgtggctggccacgacgggcgttccttgcgcagctgtgctcgacgttgtcactgaagcggg
aagggactggctgctattgggcgaagtgccggggcaggatctcctgtcatctcaccttgctcctgccgagaaagtatccatcatggctgatgca
atgcggcggctgcatacgcttgatccggctacctgcccattcgaccaccaagcgaaacatcgcatcgagcgagcacgtactcggatggaag
ccggtcttgtcgatcaggatgatctggacgaagagcatcaggggctcgcgccagccgaactgttcgccaggctcaaggcgcgcatgcccga
cggcgaggatctcgtcgtgacccatggcgatgcctgcttgccgaatatcatggtggaaaatggccgcttttctggattcatcgactgtggccggct
gggtgtggcggaccgctatcaggacatagcgttggctacccgtgatattgctgaagagcttggcggcgaatgggctgaccgcttcctcgtgcttt
acggtatcgccgctcccgattcgcagcgcatcgccttctatcgccttcttgac

Supp Fig. 7. Neomycin probe sequence. The genomic sequence amplified to generate the Southern blot Neomycin probe.

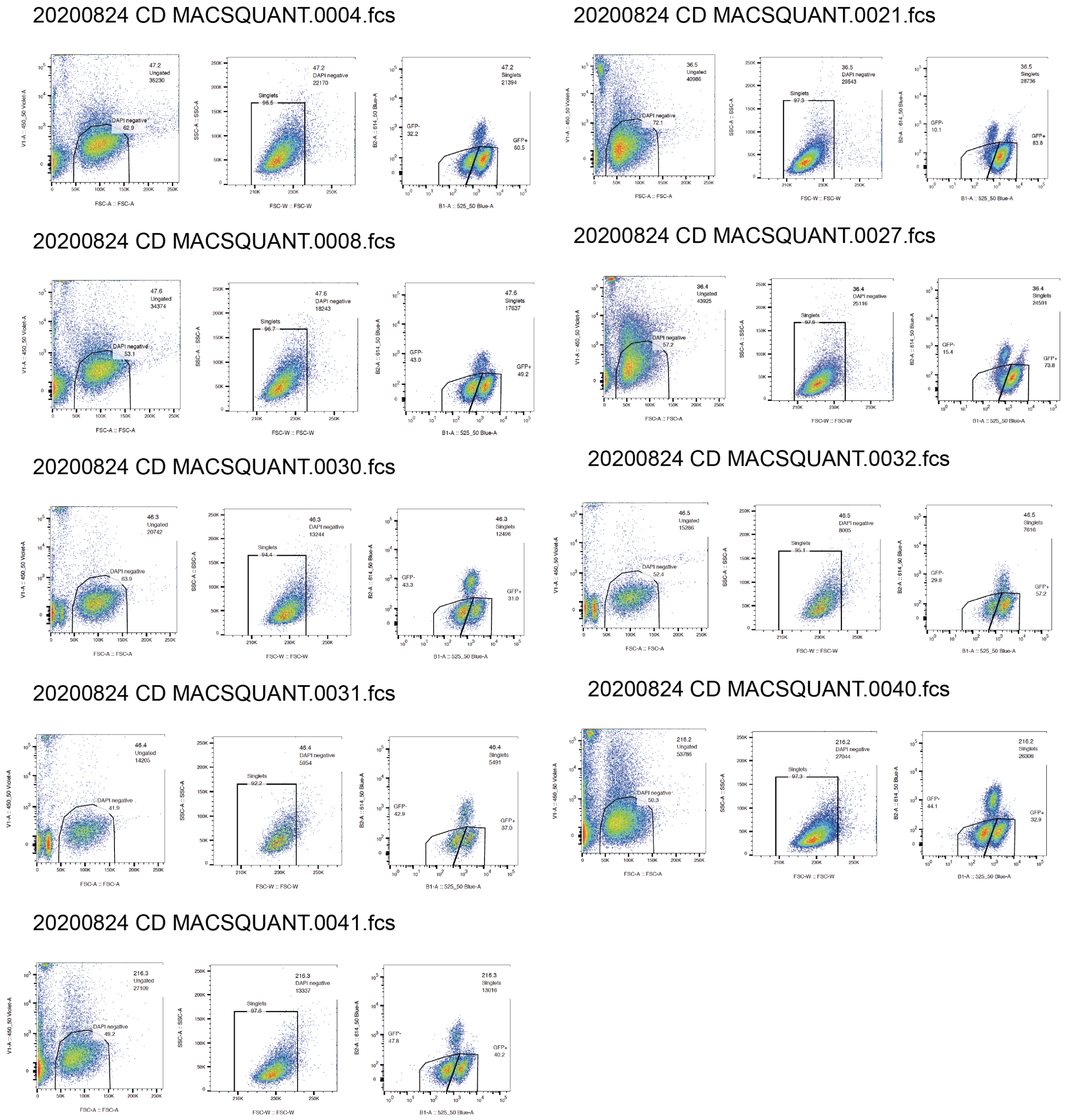

Supp Fig. 8. Flow cytometry gating on eGFP expression for determining X-inactivation status. This gating strategy is in association and reference to supplementary figure 4G.

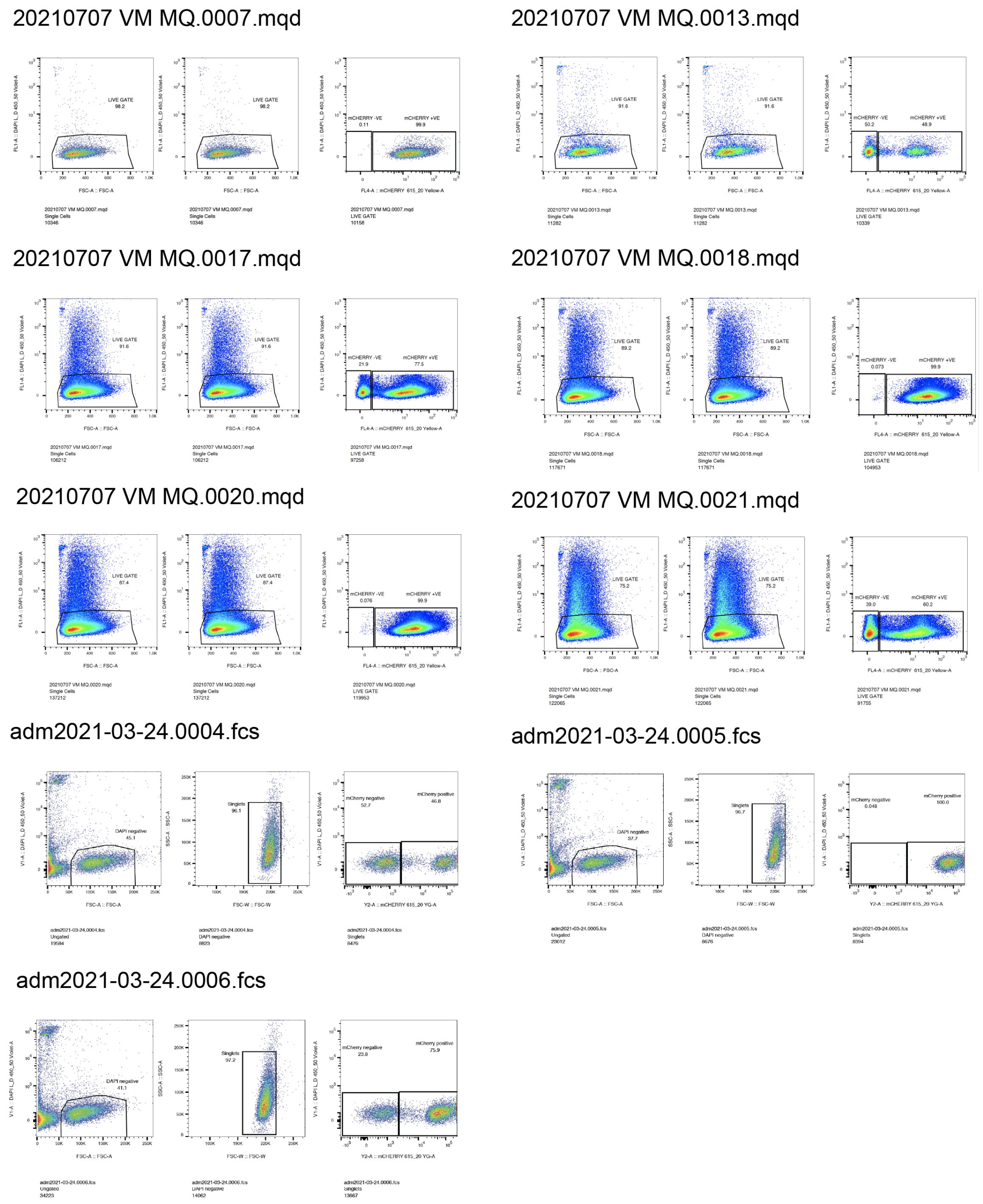

Supp Fig. 9. **Flow cytometry gating on mCherry expression for determining X-inactivation status.** This gating strategy is in association and reference to supplementary figure 2C.

Table S1. Oligonucleotide sequences		
**Primers**	**Forward**	**Reverse**
Top1 (sgRNA1, MiSeq)	TCGTCGGCAGCGTCAGATGTGTATA AGAGACAGTTGAGGCAAGGCAATGGG AT	GTCTCGTGGGCTCGGAGATGTGTATA AGAGACAGACTTTTCCCGGTCCTTATC CTT
Top1 (sgRNA2, MiSeq)	TCGTCGGCAGCGTCAGATGTGTATAAG AGACAGGAAGGAGAGACGGCAGACAC	GTCTCGTGGGCTCGGAGATGTGTATA AGAGACAGTGCAGAACATGCAAAAGC CC
Top1 (sgRNA3, MiSeq)	TCGTCGGCAGCGTCAGATGTGTATAAG AGACAGACCACAAATGGCTGAGAACTG A	GTCTCGTGGGCTCGGAGATGTGTATA AGAGACAGGGTCTGCTGCTGGTTACA GA
Atm MiSeq	TCGTCGGCAGCGTCAGATGTGTATAAG AGACAGGACTTCATCTCAGGTGGTTCC	GTCTCGTGGGCTCGGAGATGTGTATA AGAGACAGTCAGGTGGCAATGTGAAA GAC
X-Cas9 transgene	GAAACCTGGGTGTGATAGGCTT	AGGTCATGTACTGGGCACAA
Y-Cas9 transgene	AGCTGACCGGTGCAACATGAACTC	GCAGTGATGTGTCAGGTATTGGTTGT CTAC
Neomycin probe	CAGCCAATATGGGATCGGCCATTG	GTCAAGAAGGCGATAGAAGGCGATG
**sgRNAs**		**Reverse complement**
Top1 sgRNA1	CCACGAACACAAAGATCGAGAACAC	AAACGTGTTCTCGATCTTTGTGTTC
Top1 sgRNA2	CCACGGGGCTGCTGTTCACTTAGAG	AAACCTCTAAGTGAACAGCAGCCCC
Top1 sgRNA3	CCACGCGATCAAAAAGATCGTCCTC	AAACGAGGACGATCTTTTTGATCGC
Hprt	CCACTATACCTAATCATTATGCCGAGG	AAACCCTCGGCATAATGATTAGGTATA
Atm	GGTGTTCTCGACTGGTGCAC (ordered from Integrated DNA Technologies as a single guide RNA)	

**Table S2. Quantitative PCR and Digital Droplet PCR probes list**	
**Gene expression**	
Hprt	Mm03024075_m1
Gapdh	Mm99999915_g1
eGFP	Mr04097229_mr
mCherry	Mr07319438_mr
Uty	Mm00447710_m1
Eif2s3y	Mm01210630_m1
Actb	Mm02619580_g1
**Copy number**	
Tfrc	Copy number reference gene 4458370
Hprt	Mm00522878_cn
eGFP	Mr00660654_cn
Neomycin	Mr00299300_cn

**Table S3. Antibodies**	
CAS9	Novus Bio NBP2-36440
TOP1	Abcam Ab109374
TUBULIN	Sigma T9026
GAPDH	Santa Cruz Biotechnology sc-25778
MVH	Abcam Ab13840
Gamma H2AX	Millipore 05-636
SYCP3	Made In-House (published in Hirota et al, 2018)
Alexa Fluor 594 Chicken Anti-Rabbit	Thermo Fisher A21442
Alexa Fluor 488 Goat Anti-Guinea Pig	Thermo Fisher A11073
Alexa Fluor 568 Goat Anti-Mouse	Thermo Fisher A11031
Anti-mouse IgG-HRP	Santa Cruz Biotechnology sc-2005
Anti-goat IgG-HRP	Cell Signalling 7074P2

Anti-DIG-AP antibody conjugate	Roche Luminescent Detection Kit (11363514910)

Table S4. Embryo flow cytometry

Sample ID	Embryo ID	N_negative	N_positive	N_total	% Positive	Genotype
20200824 CD MACSQUANT.0027.fcs	36.4	3785	18150	21935	82.7	$X^{Cas9}Y$
20200824 CD MACSQUANT.0021.fcs	36.5	2892	24080	26972	89.3	$X^{Cas9}Y$
20200824 CD MACSQUANT.0004.fcs	47.2	6886	12941	19827	65.3	$X^{Cas9}X$
20200824 CD MACSQUANT.0007.fcs	47.5	18804	9135	27939	32.7	$X^{Cas9}X$
20200824 CD MACSQUANT.0008.fcs	47.6	7590	8680	16269	53.4	$X^{Cas9}X$
20200824 CD MACSQUANT.0030.fcs	46.3	5415	3877	9292	41.7	$X^{Cas9}X$
20200824 CD MACSQUANT.0031.fcs	46.4	2354	2032	4386	46.3	$X^{Cas9}X$
20200824 CD MACSQUANT.0032.fcs	46.5	2269	4359	6625	65.8	$X^{Cas9}X$
20200824 CD MACSQUANT.0040.fcs	216.2	11612	8645	20256	42.7	$X^{Cas9}X$
20200824 CD MACSQUANT.0041.fcs	216.3	6225	5231	11456	45.7	$X^{Cas9}X$
adm2021-03-24.0004.fcs	A3	4467	3968	8435	47	$X^{Top1}X$

adm2021-03-24.0005.fcs	A4	4	8390	8394	100	$X^{Top1}Y$
adm2021-03-24.0006.fcs	A5	3249	10377	13626	76.2	$X^{Top1}X$
20210707 VM MQ.0007.mqd	B1	11	10143	10154	99.9	$X^{Top1}Y$
20210707 VM MQ.0013.mqd	H1	5192	5054	10246	48.9	$X^{Top1}X$
20210707 VM MQ.0017.mqd	D2	21290	75390	96680	77.5	$X^{Top1}X$
20210707 VM MQ.0018.mqd	E2	77	104867	104944	99.9	$X^{Top1}Y$
20210707 VM MQ.0020.mqd	G2	91	119842	119933	99.9	$X^{Top1}Y$
20210707 VM MQ.0021.mqd	H2	35767	55269	91036	60.2	$X^{Top1}X$

Table S5. XO female whole genome sequencing (related to supplementary figure 4I)				
**Chr**	**Chr Length**	**% Of Total**	**Mapped Reads**	**% total reads mapped to each chromosome/% reads expected to map to each chromosome**
chr1	195471971	7.17186826	459	93.24018086
chr2	182113224	6.68173572	506	110.3275433
chr3	160039680	5.871857205	374	92.79377475
chr4	156508116	5.742284092	410	104.0212114
chr5	151834684	5.570815833	412	107.745996
chr6	149736546	5.493835132	437	115.8853508
chr7	145441459	5.336248354	456	124.4948923
chr8	129401213	4.747731593	376	115.3783732
chr9	124595110	4.571395634	438	139.5879813
chr10	130694993	4.795200392	324	98.43759038
chr11	122082543	4.47920953	323	105.0567276
chr12	120129022	4.407534828	318	105.1124339
chr13	120421639	4.418270948	303	99.9109316
chr14	124902244	4.582664383	339	107.7716101
chr15	104043685	3.817363678	253	96.55609858
chr16	98207768	3.603243834	228	92.18577689
chr17	94987271	3.485083772	266	111.1965057
chr18	90702639	3.327880588	203	88.86915048
chr19	61431566	2.253924673	156	100.834216
chrM	16299	0.00059801	0	0
chrX	171031299	6.275139799	262	60.82759013
chrY	91744698	3.366113741	21	9.088939931
Total	2725537669		6864	
