# Supplementary material for: CRISPR-Cas9 effectors facilitate generation of single-sex litters and sex-specific phenotypes
Source: Nat Commun. 2021 Dec 3;12:6926. doi: 10.1038/s41467-021-27227-2 (PMC8642469; doi:10.1038/s41467-021-27227-2)
Supplement: Supplementary file 2 — Reporting Summary [file 41467_2021_27227_MOESM2_ESM.pdf]

nature portfolio

Corresponding author(s): James M. A. Turner
Peter J. I. Ellis

Last updated by author(s): Oct 29, 2021

Reporting Summary

Nature Portfolio wishes to improve the reproducibility of the work that we publish. This form provides structure for consistency and transparency in reporting. For further information on Nature Portfolio policies, see our Editorial Policies and the Editorial Policy Checklist.

Statistics

For all statistical analyses, confirm that the following items are present in the figure legend, table legend, main text, or Methods section.

n/a	Confirmed	
☐	☒	The exact sample size (*n*) for each experimental group/condition, given as a discrete number and unit of measurement
☐	☒	A statement on whether measurements were taken from distinct samples or whether the same sample was measured repeatedly
☐	☒	The statistical test(s) used AND whether they are one- or two-sided<br>*Only common tests should be described solely by name; describe more complex techniques in the Methods section.*
☒	☐	A description of all covariates tested
☒	☐	A description of any assumptions or corrections, such as tests of normality and adjustment for multiple comparisons
☐	☒	A full description of the statistical parameters including central tendency (e.g. means) or other basic estimates (e.g. regression coefficient) AND variation (e.g. standard deviation) or associated estimates of uncertainty (e.g. confidence intervals)
☐	☒	For null hypothesis testing, the test statistic (e.g. *F*, *t*, *r*) with confidence intervals, effect sizes, degrees of freedom and *P* value noted<br>*Give P values as exact values whenever suitable.*
☒	☐	For Bayesian analysis, information on the choice of priors and Markov chain Monte Carlo settings
☒	☐	For hierarchical and complex designs, identification of the appropriate level for tests and full reporting of outcomes
☒	☐	Estimates of effect sizes (e.g. Cohen's *d*, Pearson's *r*), indicating how they were calculated

Our web collection on statistics for biologists contains articles on many of the points above.

Software and code

Policy information about availability of computer code

Data collection	No commercial, open source or custom code was used to collect data.
Data analysis	MiSeq libraries were demultiplexed and fastq files collapsed using FastX Toolkit (v0.0.13). Reads were aligned to mm10 using BurrowsWheeler Alignment Tool (BWA, v 0.7.170). Analysis was performed using R package CrispRVariants (v 1.14.0). CRISPR-Cas9 MiSeq scripts are deposited on GitHub (https://github.com/jzohren/crispr-miseq). The DOI for the GitHub repository is: 10.5291/zenodo.5557228.<br>Whole genome sequencing libraries (using Oxford Nanopore Technologies sequencing) were basecalled using ONT-Guppy v3.2. Data was mapped using minimap2 (v2.17) and SAMtools (v1.9), and analysed using base R (v4.0.2) and Excel.<br>Flow cytometry data was analysed using FlowJo v10.7 software. The digital droplet qPCR (ddPCR) results were analysed with QuantaSoft (v1.7.4.0917). Ovary section images were analysed using QuPath (v0.2.3) (Open Source) software.

For manuscripts utilizing custom algorithms or software that are central to the research but not yet described in published literature, software must be made available to editors and reviewers. We strongly encourage code deposition in a community repository (e.g. GitHub). See the Nature Portfolio guidelines for submitting code & software for further information.

Data

Policy information about availability of data

All manuscripts must include a data availability statement. This statement should provide the following information, where applicable:

- Accession codes, unique identifiers, or web links for publicly available datasets
- A description of any restrictions on data availability
- For clinical datasets or third party data, please ensure that the statement adheres to our policy

The data that supports the findings of this study are available from the corresponding author upon reasonable request. MiSeq and low-pass Whole Genome

Sequencing read counts are available in the Source Data file. Mouse reference genome mm10 was used for mapping the reads in this study.

Field-specific reporting

Please select the one below that is the best fit for your research. If you are not sure, read the appropriate sections before making your selection.

- [x] Life sciences
- [ ] Behavioural & social sciences
- [ ] Ecological, evolutionary & environmental sciences

For a reference copy of the document with all sections, see nature.com/documents/nr-reporting-summary-flat.pdf

Life sciences study design

All studies must disclose on these points even when the disclosure is negative.

Sample size	The minimum number of pups was analysed in order to establish whether there was a sex skew from an expected 50:50 male:female ratio. Sample size was considered to be sufficient number of pups to accurately make the assumption that single-sex litters could not be down to random chance. For all expression data, such as qPCR, samples were tested in triplicate. All western blots were performed a minimum of three times. All embryos were analysed at a minimum of n=3 biological replicates. All in vivo experiments used a minimum of two independent animals. Legends have been edited accordingly.
Data exclusions	No data was excluded.
Replication	All attempts at replication were successful. The number of replicates is noted in all figure legends.
Randomization	No covariates are present in this study. The purpose of the study is to establish if all male or female litter can be created. The nature of the experiments does require randomisation.
Blinding	Blinding was not relevant to the study as genotypes were known in order to set up relevant breedings. The expected output of single-sex litters (experimental) or normal Mendelian frequency (control) was unknown to Biological Research Facility staff.

Reporting for specific materials, systems and methods

We require information from authors about some types of materials, experimental systems and methods used in many studies. Here, indicate whether each material, system or method listed is relevant to your study. If you are not sure if a list item applies to your research, read the appropriate section before selecting a response.

Materials & experimental systems

n/a	Involved in the study	
[ ]	[x]	Antibodies
[ ]	[x]	Eukaryotic cell lines
[x]	[ ]	Palaeontology and archaeology
[ ]	[x]	Animals and other organisms
[x]	[ ]	Human research participants
[x]	[ ]	Clinical data
[x]	[ ]	Dual use research of concern

Methods

n/a	Involved in the study	
[x]	[ ]	ChIP-seq
[ ]	[x]	Flow cytometry
[x]	[ ]	MRI-based neuroimaging

Antibodies

Antibodies used	Cas9 (Novus Bio NBP2-36440), Topl (Abeam Ab109374), Tubulin (Sigma T9026), Gapdh (Santa Cruz Biotechnology sc-25778), MVH (Abeam Ab13840), Gamma H2AX (Millipore 05-636), SYCP3 (Made in house), Alexa Fluor 488 (Thermo Fisher A11073), Alexa Fluor 568 (Thermo Fisher A11031), Alexa Fluor 594 (Thermo Fisher A21442), Anti-mouse lgG-HRP (Santa Cruz Biotechnology sc-2005), Anti-rabbit lgG-HRP (Cell Signalling 7074P2), Anti-DIG (Roche luminescent detection kit 11363514910).
Validation	Cas9 antibody validated for WB, IB, ICC/IF, IHC and reactivity with mouse, bacteria, chicken, fungi according to manufacturer's website with citations (https://www.novusbio.com/products/crispr-cas9-antibody-7a9-3a3_nbp2-36440). Topl antibody validated for WB, IHC, Flow Cyt, ICC and reactivity with mouse, rat, human according to manufacturer's website (https://www.abcam.com/topoisomerase-i-antibody-epr5375-ab109374.html). MVH antibody validated for WB, ICC/IF, IHC and reactivity with mouse, human by Abcam (https://www.abcam.com/ddx4--mvh-antibody-ab13840.html) and used in previous publications e.g. PMID: 29114052. Gamma H2AX antibody validated for ICC, IF, WB, ChIP, IHC and reactivity with vertebrate according to manufacturer's website (https://www.merckmillipore.com/GB/en/product/Anti-phospho-Histone-H2A.X-Ser139-Antibody-clone-JBW301,MM_NF-05-636?ReferrerURL=https%3A%2F%2Fwww.google.com%2F). Gamma H2AX and SYCP3 antibody used in previous publications for IF in mouse e.g. PMID: 30393076. Tubulin antibody validated for IF and IB and reactivity with mouse, human, bovine, amphibian, yeast, fungi according to manufacturer's information (https://www.sigmaaldrich.com/deepweb/assets/sigmaaldrich/product/documents/149/523/t9026dat.pdf). Gapdh antibody validated for WB and reactivity with mouse, human according to manufacturer's website (https://www.scbt.com/p/gapdh-antibody-fl-335).

Eukaryotic cell lines

Policy information about cell lines

Cell line source(s)	Rosa26-Cas9 mouse embryonic stem cells were derived from blastocysts from the transgenic mouse line, described in Platt et al, (Cell), 2014.
Authentication	None of the cell lines were authenticated.
Mycoplasma contamination	All cell lines were tested for Mycoplasma and were negative.
Commonly misidentified lines (See ICLAC register)	No commonly misidentified cell lines were used.

Animals and other organisms

Policy information about studies involving animals; ARRIVE guidelines recommended for reporting animal research

Laboratory animals	Wildtype or control mice refers to C57BL/6J mice. Hll(Topl) transgenic mice were generated and maintained on a C57BL/6J background. X(Cas9) and Y(Cas9) mice were generated in C57BL/6N stem cells, and transgenic mice were maintained on a C57BL/6J background. X(Topl) mice were generated on in 129/Ola embryonic stem cells prior to generating a stable transgenic line maintained on a C57BL/6J background. All mouse lines (Mus musculus) were maintained with appropriate care according to the United Kingdom Animal Scientific Procedures Act (1986), UK Home Office, and the ethics guidelines of the Francis Crick Institute. All mice were kept in individually ventilated cages (IVC), with constant access to food, automatic watering systems, and air management systems which control air flow, temperature (20-22 C) and humidity (49-62 %). The mice were kept under a 12 hour light and 12 hour dark cycle. The mouse lines were checked daily and maintained in specific pathogen free (SPF) conditions. Sufficient nesting material and environmental enrichment was provided. Transgenic lines produced by GenOway were cared for according to ethics guidelines and protocols, and by Charles River France, prior to shipment to the Francis Crick Institute. All mice were of sexual maturity age when they were set up in experimental and control breedings. Pups born were genotyped at 2 weeks old by standard ear biopsy genotyping. 6 – 10-week old female and 8 – 14-week male mice were used for matings. Please see legend for more information regarding the sexes used.
Wild animals	The study did not involve wild animals.
Field-collected samples	The study did not involve samples collected from the field.
Ethics oversight	All mouse lines were maintained with appropriate care according to the United Kingdom Animal Scientific Procedures Act (1986), UK Home Office and ethics guidelines of the Francis Crick Institute. All procedures were performed under the Francis Crick Institute Genetic Modification Service, or James Turner's project Home Office license.

Note that full information on the approval of the study protocol must also be provided in the manuscript.

Flow Cytometry

Plots

Confirm that:

- [x] The axis labels state the marker and fluorochrome used (e.g. CD4-FITC).
- [x] The axis scales are clearly visible. Include numbers along axes only for bottom left plot of group (a 'group' is an analysis of identical markers).
- [x] All plots are contour plots with outliers or pseudocolor plots.
- [x] A numerical value for number of cells or percentage (with statistics) is provided.

Methodology

Sample preparation	E9.5-E12.5 mouse embryos were prepared according to Dupont et al, (2017). Embryos were dissected out of the uterus, and the placenta and head removed. Embryo cells were dissociated by addition of 0.25% Trypsin/EDT A (37 degrees C, 10 mins) before manual dissociation using a needle and syringe. 20% FBS in PBS was added to the cell suspension, centrifuged, resuspended in 2% FBS in PBS and filtered (40uM) before performing flow cytometry.
Instrument	MACS Quant VYB
Software	FlowJo (v10. 7)
Cell population abundance	XCas9Y (82.7% GFP positive)<br>XCas9Y (89.3% GFP positive)<br>XCas9X (65.3% GFP positive)<br>XCas9X (32.7% GFP positive)

XCas9X (53.4% GFP positive)
XCas9X (41.7% GFP positive)
XCas9X (46.3% GFP positive)
XCas9X (65.8% GFP positive)
XCas9X (42.7% GFP positive)
XCas9X (45.7% GFP positive)
XToplX (47% mCherry positive)
XToplY (100% mCherry positive)
XToplX (76.2% mCherry positive)

Gating strategy	Using FSC/SSCg ating, dead cells and doublets/clumps of cells were removed. Alive single cells were then analysed for expression of reporters (either GFP or mCherry, depending on the transgene), and non-reporter cells, in individual embryos.

☒ Tick this box to confirm that a figure exemplifying the gating strategy is provided in the Supplementary Information.
